# EPB41L family serves as a prognostic biomarker for kidney renal clear cell carcinoma

**DOI:** 10.1080/19336918.2026.2624964

**Published:** 2026-02-04

**Authors:** Gonglin Tang, Kai Sun, Guixin Ding, Jitao Wu, Jian Ma, Hongwei Zhao

**Affiliations:** a Department of Urology, The Affiliated Yantai Yuhuangding Hospital of Qingdao University, Yantai, Shandong, China; b Urology Department, Shandong Provincial Hospital, Shandong University, Jinan, China

**Keywords:** EPB41L family, immune infiltration, kidney renal clear cell carcinoma, prognostic biomarker

## Abstract

EPB41L1-5 is known to maintain cell morphology and signal transduction, with evidence suggesting it can inhibit tumor progression. However, its role in kidney renal clear cell carcinoma (KIRC) is not fully understood. This study evaluated EPB41L1-5’s prognostic value in KIRC using bioinformatics methods and validation through qPCR, immunohistochemistry, and cell functional experiments. The results demonstrated a decreased expression of EPB41L in KIRC tissue compared to normal renal tissue, correlating with lower survival rates. Low EPB41L expression was also associated with overall survival in KIRC. Additionally, EPB41L was found to be involved in extracellular matrix regulation, G protein-coupled receptor ligand binding, and multiple immune cell infiltrations. In addition, their elevated methylation levels are associated with poor prognosis in KIRC patients. Overall, EPB41L family is a potential molecular marker for predicting KIRC prognosis, offering insights for therapeutic development.

## Introduction

Kidney Renal Clear Cell Carcinoma (KIRC) is a type of kidney cancer that originates in the cells lining the small tubes within the kidney. KIRC is the most common type of kidney cancer, accounting for approximately 70–80% of all cases. It is a highly aggressive cancer that can spread to other parts of the body [1,2]. The precise etiology of KIRC remains incompletely understood, yet several risk factors have been identified, including smoking, obesity, high blood pressure, and a familial history of kidney cancer [2]. Treatment options for KIRC include surgery to remove the affected kidney or a portion of it, radiation therapy, chemotherapy, or targeted therapy [3]. KIRC, particularly in advanced stages, remains a formidable therapeutic challenge. Current research efforts focus on developing novel treatment strategies and improving early diagnostic approaches to enhance patient outcomes.

The EPB41L gene family, also known as the erythrocyte membrane protein band 4.1-like (EPB41L) family, comprises a group of genes that encode proteins involved in cellular cytoskeletal organization, signal transduction, and membrane transport [4,5]. These proteins play a crucial role in maintaining cellular morphology, signal transduction, cell adhesion, and cell motility, among other biological processes. The family consists of 6 members: EPB41L1, EPB41L2, EPB41L3, EPB41L4A, EPB41L4B and EPB41L5. This protein is also associated with the occurrence and development of tumors and is considered a tumor suppressor. Mutations in the EPB41L gene are related to the occurrence and development of various diseases, such as cataracts, KIRC, breast cancer, and colon cancer [6–8]. In the era of precision oncology for KIRC, therapeutic decisions increasingly rely on tumor-intrinsic and microenvironmental features rather than histology alone [9].

Bioinformatics tools are employed to interrogate the whole-genome sequencing data of urological neoplasms, aiming to identify tumor-specific genetic aberrations, copy number variations, and structural variants [10]. Transcriptomic analyses have elucidated alterations in gene expression within tumors, aiding researchers in deciphering the molecular underpinnings of neoplastic processes [11,12]. Furthermore, artificial intelligence and machine learning technologies are harnessed for pattern recognition, predictive model development, and the analysis of voluminous datasets, advancing the discovery of novel biomarkers and therapeutic approaches. The role of bioinformatics in diverse domains of medical research is poised for continued expansion [13,14].

In this study, we investigated the functional roles of the EPB41L family in KIRC. Leveraging data from The Cancer Genome Atlas (TCGA) database, we examined the expression profiles of EPB41L1-5 and evaluated their prognostic significance in KIRC. We first compared EPB41L expression levels between tumor and adjacent normal kidney tissues from KIRC patients. Subsequently, we analyzed the associations between EPB41L expression and clinicopathological characteristics using univariate and multivariate Cox proportional hazards regression analyses. Based on these findings, we constructed a prognostic nomogram and validated its predictive performance. To elucidate the underlying biological mechanisms of EPB41L function, we performed Gene Ontology (GO) enrichment analysis and gene set enrichment analysis (GSEA). Considering the known association between EPB41L and methylation, we also investigated its relationship with mutations, immune infiltration, and methylation [15–17]. Lastly, we validated the expression of EPB41L in clinical specimens from KIRC patients and renal cancer cell lines, providing a comprehensive analysis of the connection between EPB41L and tumor development.

## Materials and methods

### Clinical data from TCGA databases

We obtained gene expression data, including HTSeq-Counts and HTSeq-FPKM, phenotype data, and extensive clinicopathological information for TCGA-KIRC through the UCSC Xena platform. The sequence data was sourced from the Illumina HiSeq_RNA_Seq platform. Following this, the HTSeq-FPKM gene expression data was converted to TPM (transcripts per million reads) format for further analyses.

### Low-expression of EPB41L in samples with KIRC

The expression levels of EPB41L were compared between tumor tissues and their corresponding normal tissues in patients diagnosed with KIRC. To investigate the correlations among EPB41L1-5 levels in KIRC, the corrplot R package (version 0.85) and pROC R package (version 1.18) were employed.

### Correlation between EPB41L and survival

Kaplan-Meier survival curves were generated using the survival R package (version 0.13) to evaluate the prognostic value of EPB41L1-5 expression. With patients stratified into high- and low-expression groups based on the median expression value of each EPB41L gene through the TCGA database; statistical significance was assessed using the log-rank test, with a threshold of *p* < .05, and follow-up time was measured in months from diagnosis to death or last clinical follow-up.

### Development and assessment of a nomogram and prognostic model

Cox regression analysis was employed for prognostic model development, with univariate analysis including variables significant at *p* < .05 for multivariate analysis, which computed hazard ratios (HR) with 95% confidence intervals, and the proportional hazards assumption was verified using Schoenfeld residuals test. The nomogram was constructed using the rms R package (version 6.0) and Hmisc R package (version 4.4) to predict 1-, 3-, and 5-year overall survival probabilities, followed by model validation through C-index calculation with 1000 bootstrap replicates, calibration plot generation via 1000 bootstrap resampling iterations.

### Functional enrichment analysis

For the identification of co-expressed genes with EPB41L1-5, we utilized GEPIA2.0 (http://gepia2.cancer-pku.cn/). Significant co-expression pairs were defined based on the following criteria: a Pearson correlation coefficient ∣R∣ > 0.4 and a statistical significance of *p* < .001. To focus on strong positive associations, the top 100 positively correlated genes for each EPB41L member were selected for further analysis. Subsequently, Gene Ontology (GO) enrichment analysis of these co-expressed gene sets was performed using Metascape (version 3.5, https://metascape.org/) with the whole human genome serving as the background. Statistically significant GO terms were filtered based on a p-value < .01, a minimum enrichment score > 1.5, and a minimum gene count > 3, with multiple testing correction applied using the Benjamini-Hochberg false discovery rate (FDR) method.

### Gene set enrichment analysis

To identify differentially expressed genes, we performed RNA-seq data analysis using the DESeq2 R package (version 1.28) with median of ratios normalization. Genes with an absolute log2 fold change greater than 1 and an adjusted p-value below 0.05 were considered statistically significant [18,19]. For gene set enrichment analysis, we employed both the clusterProfiler (version 4.0.5) [20]. The objective of this analysis was to identify statistically significant disparities in pathways between populations with high and low expression levels of EPB41L1-5 in KIRC. The expression level of EPB41L1-5 was used as the phenotype label. Pathway terms were considered significantly enriched if they exhibited an adjusted P-value of less than 0.05 and an FDR q-value of less than 0.25.

EPB41L1-5 mutations and prognosis cBioPortal serves as an invaluable resource for exploring, visualizing, and analyzing multidimensional cancer genome data [21]. In our research, we conducted a thorough examination of the genomic profiles for EPB41L1-5, setting a z-score threshold of ±1.8. We meticulously assessed the genetic mutations in EPB41L1-5 and their correlation with overall survival (OS).

### Evaluation of immune infiltration in relation to EPB41L1-5 expression through ssGSEA analysis

In order to assess the associations between EPB41L1-5 and the levels of immune cell infiltration, the ssGSEA (single-sample Gene Set Enrichment Analysis) technique was employed utilizing the GSVA R package (version 1.40). The comparative levels of 24 types of immune cells infiltrating tumors were assessed based on immunocyte signatures, which incorporated 714 genes for individual tissue sample prediction. By referencing immunocyte signature genes from the literature, a relative enrichment score was calculated for each immunocyte based on the gene expression profile of individual tumor samples [22]. The association between immune infiltration scores and gene expression was evaluated using Spearman correlation analysis, with statistical significance defined as *p* < .05.

### The correlation between the expression of EPB41L1-5 and methylation

Research suggests that EPB41L is linked to methylation. Consequently, we carried out an additional analysis on the association between EPB41L1-5 and methylation. The Spearman correlation coefficient was calculated to assess the correlation between EPB41L1-5 and methylation levels in TCGA-KIRC. MethSurv was employed for this analysis.

### Tissue sample acquisition

Between 2021 and 2022, a total of 47 patients who underwent radical or partial nephrectomy at the urology department of Qingdao University Affiliated Yantai Yuhuangding Hospital generously donated KIRC tissues along with corresponding normal renal tissues. Immediately after surgery, the specimens were submerged in liquid nitrogen and subsequently stored in a − 80°C freezer for later analysis. Prior informed consent was obtained from all participants, and the study was ethically approved by the esteemed ethics committee of Qingdao University Affiliated Yantai Yuhuangding Hospital.

### Cell purchase and culture

The KIRC cell lines, including 786-O, 769-P, ACHN, Caki-2, and A498, along with the human normal renal proximal convoluted tubular cell line HK-2, are available for purchase from the Cell Bank of the Chinese Academy of Sciences. These KIRC cell lines were grown in RPMI 1640 medium, whereas HK-2 was cultured in DMEM (BI, Israel). Both media contained 10% Fetal Bovine Serum (FBS). All cells were maintained in a humidified incubator set at 37 degrees Celsius and 5% carbon dioxide.

### RNA extraction, reverse transcription, and qRT-PCR quantification

RNA was extracted from freshly frozen tissues or KIRC cell lines utilizing the RNA Extraction Kit (Qiagen, China). This was succeeded by reverse transcription with the Evo M-MLV RT Mix Kit (Accurate Biology, China). qPCR was then conducted using the SYBR® Green Premix Pro Taq HS qPCR Kit and Rox Reference Dye (Accurate Biology, China), with GAPDH serving as the internal reference gene, and the relative expression level of the target gene was determined using the 2^−ΔΔCT^ calculation method. Each experiment was conducted in triplicate [23].

### Immunohistochemical

Immunohistochemical staining was conducted utilizing a Roche Bench Mark GX semi-automatic machine for the detection of cancerous and adjacent non-cancerous tissues. Paraffin-embedded samples were sliced at 0.4 mm intervals, and two consecutive sections were cut and heated at 65°C for half an hour. Subsequently, the thin sections underwent HE staining to enhance tissue visualization. Under high magnification, ten random fields of view were selected, and a scoring system was applied based on staining intensity: light yellow cells received a score of 1, yellow-stained cells a 2, and brown-stained cells a 3. The proportion of positively stained cells was assessed as follows: less than 10% (score 0), 11–25% (score 1), 26–50% (score 2), 51–75% (score 3), and over 75% (score 4). The final immunohistochemical score was determined by multiplying these two scores.

### Western blot

The cells were lysed in RIPA buffer (Servicebio, China) containing 1% protease inhibitor for 30 minutes, followed by further sonication on ice using a sonicator at approximately 15% power for 10–15 seconds. The resulting protein supernatant was obtained after centrifugation. The supernatant was then mixed with 5× protein loading buffer (Coolaber, China) and heated at 100°C for 10 minutes for sample preparation. SDS-PAGE was performed for electrophoresis, and the proteins were transferred onto a PVDF membrane (Millipore, USA). The membrane was blocked with TBST buffer containing 5% skim milk for 2 hours at room temperature, and then incubated with various primary antibodies overnight at 4°C on a shaker. The membrane was subsequently incubated with secondary antibodies at room temperature for 1 hour. The ECL substrate solution A and B were mixed in equal proportions to prepare the developing solution, which was evenly dropped onto the PVDF membrane and visualized using a protein imaging system. The antibodies used were as follows: rabbit polyclonal EPB41L2 antibody (Abmart, China, Cat No. TP72110S, 1:1000), mouse monoclonal GAPDH antibody (Proteintech, Cat No. 60,004–1-Ig, China, 1:1000), HRP-conjugated goat anti-rabbit secondary antibody (Proteintech, Cat No. SA00001-2, China, 1:5000), and HRP-conjugated goat anti-mouse secondary antibody (Proteintech, Cat No. SA00001-1, China, 1:5000).

### Transfection

EPB41L2 overexpression and negative control (NC) lentiviral vectors were synthesized by OBiO Technology Company (China), and subsequently transfected into KIRC cell lines using polybrene.

### CCK‑8 assay

ACHN and 769-P cells, which had been transfected with either NC or EPB41L2, were plated in 96-well plates at a density of 2 × 10^3^ cells per well. At 0, 24, 48, 72, and 96 hours, CCK-8 reagent was added. Following a 2-hour incubation at 37°C, cell absorbance was measured at 450 nm using a spectrophotometer.

### Wound healing assay

The cells were seeded at a density of 2 × 10^6^ per well in 6-well plates and allowed to adhere for 12 hours. A sterile 1000 μl pipette tip was used to create horizontal and vertical scratches. After cleaning the orifice plate with phosphate buffer, images were taken immediately (at the 0-hour time point) using a microscope. Subsequently, 2 ml of RPMI 1640 complete medium was added to each well for incubation, and images were captured again at the same location after 12 hours. The extent of wound closure was employed as a measure of cell migration [24].

### Transwell invasion assay

We used a transwell plate with an 8 μm polycarbonate membrane to evaluate cell invasion. A 65 μl Matrigel matrix was applied to the upper layer of the membrane and incubated for 2 hours. Cells, resuspended in serum-free medium at a density of 5 × 10^4^ cells per well, were then seeded onto the Matrigel matrix, while complete medium was added to the lower compartment. After incubation for 24 to 48 hours, the cells were fixed with methanol, stained with 0.5% Giemsa stain (Perfemiker, China), and visualized and quantified under a microscope.

### Statistical analysis

Statistical analyses were conducted using IBM SPSS Statistics (version 25.0) and R (version 4.2.1). The Wilcoxon rank-sum test was employed to compare EPB41L1-5 expression between KIRC and normal groups. The Wilcoxon signed-rank test was used to assess the relationship between clinical pathological parameters and EPB41L1-5 expression levels. Cox regression analysis was performed to evaluate the association of clinical pathological parameters with OS. Both univariate and multivariate Cox regression models were used to explore correlations between expression levels and clinicopathological parameters. Hazard ratios (HR) with 95% confidence intervals (CI) were calculated to assess the risk associated with individual factors. All tests were two-sided, with *p* values less than 0.05 considered statistically significant.

## Results

### Clinical characteristics

Data were collected from TCGA (Table 1, Supplementary Table S1), including gene expression and clinical information. This dataset encompasses patient characteristics such as age, TNM stage, and EPB41L1-5 gene expression.

**Table 1.** TCGA KIRC patient characteristics.

Characteristic	Low expression of EPB41L1	High expression of EPB41L1	p	Low expression of EPB41L2	High expression of EPB41L2	p	Low expression of EPB41L3	High expression of EPB41L3	p
n	269	270		269	270		269	270	
T stage, n (%)			<0.001			0.313			0.741
T1	103 (19.1%)	175 (32.5%)		129 (23.9%)	149 (27.6%)		134 (24.9%)	144 (26.7%)	
T2	48 (8.9%)	23 (4.3%)		36 (6.7%)	35 (6.5%)		34 (6.3%)	37 (6.9%)	
T3	108 (20%)	71 (13.2%)		99 (18.4%)	80 (14.8%)		95 (17.6%)	84 (15.6%)	
T4	10 (1.9%)	1 (0.2%)		5 (0.9%)	6 (1.1%)		6 (1.1%)	5 (0.9%)	
N stage, n (%)			0.181			1.000			0.488
N0	116 (45.1%)	125 (48.6%)		119 (46.3%)	122 (47.5%)		120 (46.7%)	121 (47.1%)	
N1	11 (4.3%)	5 (1.9%)		8 (3.1%)	8 (3.1%)		6 (2.3%)	10 (3.9%)	
M stage, n (%)			<0.001			0.215			0.538
M0	199 (39.3%)	229 (45.3%)		211 (41.7%)	217 (42.9%)		211 (41.7%)	217 (42.9%)	
M1	54 (10.7%)	24 (4.7%)		45 (8.9%)	33 (6.5%)		42 (8.3%)	36 (7.1%)	
Age, median (IQR)	62 (52, 71)	60 (52, 69)	0.152	62.39 ± 12.08	58.87 ± 11.87	<0.001	61.81 ± 11.73	59.44 ± 12.35	0.023

### Expression status of EPB41L in KIRC

**Figure 1.**
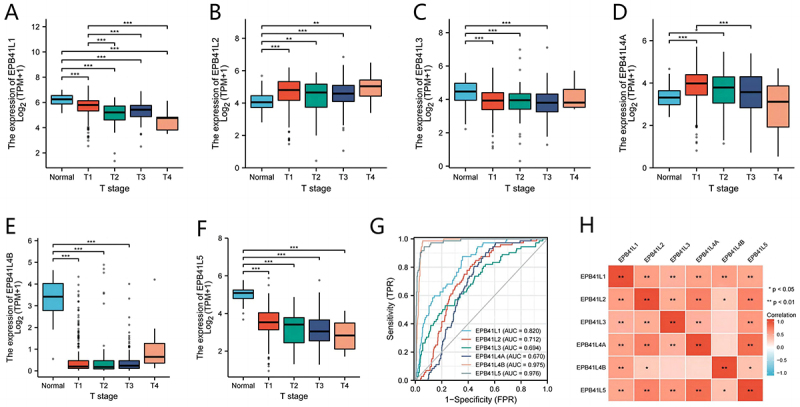
EPB41L1-5 expression levels in KIRC from TCGA data. (A-F) the expression levels of EPB41L1-5 in KIRC and normal tissue, their expression is generally high in normal tissues (G) Receiver operating characteristic analysis (ROC) of EPB41L1-5 in KIRC. (H) the correlation between EPB41L1-5 members. *p < .05, **p < .01, ***p < .001.

The Wilcoxon rank-sum test revealed that EPB41L1-5 expression in KIRC tissue was markedly lower compared to normal tissue (*p* < .001) (Figure 1(A–F)). This substantial downregulation of EPB41L1-5 in KIRC indicates its potential role in KIRC development (*p* < .001). A significant correlation was observed between the gene levels of EPB41L1-5 (Figure 1(G)). The AUC values for EPB41L1-5 were 0.820, 0.712, 0.694, 0.670, 0.975, and 0.976, respectively (Figure 1(H)).

### Association between EPB41L and survival

**Figure 2.**
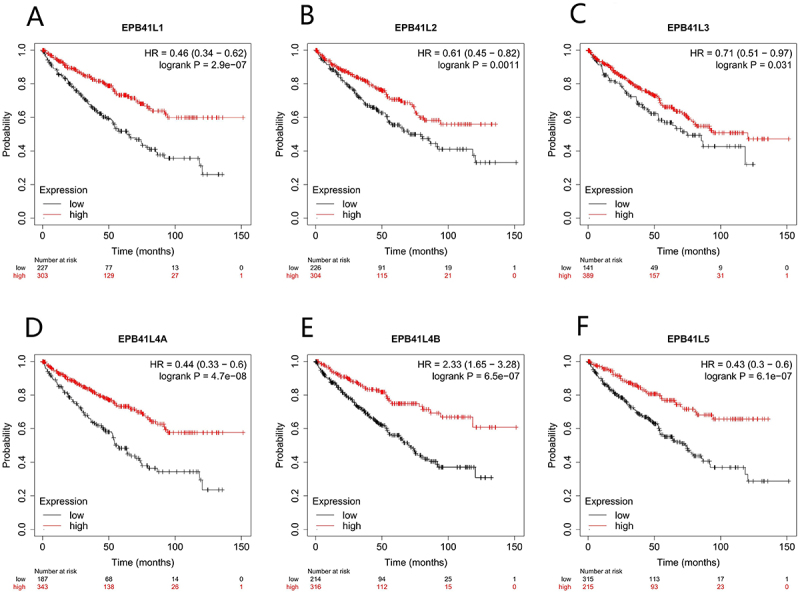
The prognostic value of EPB41L1-5 expression in KIRC. (A–F) survival curves of OS from TCGA data.

We have observed that patients with low expression levels of EPB41L1-5 exhibit significantly lower OS compared to those with high expression levels (Figure 2(A–F), Figure S1, S2). We then used a univariate Cox regression model to evaluate prognostic factors in KIRC (Table 2, Supplementary Table S2). This analysis showed that lower EPB41L expression levels were linked to reduced OS. Following this, a multivariate Cox regression analysis was performed, which revealed that clinical T stage was independently associated with OS.

**Table 2.** Univariate and multivariate Cox regression model of prognosis for EPB41L1-3 in patients with KIRC.

Characteristics	Total(N)	Univariate analysis		Multivariate analysis	
		Hazard ratio (95% CI)	P value	Hazard ratio (95% CI)	P value
Gender	541				
Female	187	Reference			
Male	354	0.924 (0.679 - 1.257)	0.613		
Race	534				
Asian&Black or African American	65	Reference			
White	469	1.233 (0.685 - 2.221)	0.485		
Age	541				
≤60	269	Reference		Reference	
> 60	272	1.791 (1.319 - 2.432)	** <0.001**	1.583 (1.032 - 2.428)	**0.036**
Pathologic T stage	541				
T1&T2	350	Reference		Reference	
T3&T4	191	3.210 (2.373 - 4.342)	** <0.001**	1.582 (0.694 - 3.606)	0.275
Pathologic N stage	258				
N0	242	Reference		Reference	
N1	16	3.422 (1.817 - 6.446)	** <0.001**	1.495 (0.740 - 3.017)	0.262
Pathologic M stage	508				
M0	429	Reference		Reference	
M1	79	4.401 (3.226 - 6.002)	** <0.001**	2.711 (1.600 - 4.594)	** <0.001**
Pathologic stage	538				
Stage I&Stage II	332	Reference		Reference	
Stage III&Stage IV	206	3.910 (2.852 - 5.360)	** <0.001**	1.161 (0.460 - 2.934)	0.752
Histologic grade	533				
G1&G2	250	Reference		Reference	
G3&G4	283	2.665 (1.898 - 3.743)	** <0.001**	1.620 (0.990 - 2.652)	0.055
EPB41L1	541				
Low	270	Reference		Reference	
High	271	0.420 (0.304 - 0.578)	** <0.001**	0.551 (0.352 - 0.861)	**0.009**
Gender	541				
Female	187	Reference			
Male	354	0.924 (0.679 - 1.257)	0.613		
Race	534				
Asian&Black or African American	65	Reference			
White	469	1.233 (0.685 - 2.221)	0.485		
Age	541				
≤60	269	Reference		Reference	
> 60	272	1.791 (1.319 - 2.432)	** <0.001**	1.580 (1.027 - 2.429)	**0.037**
Pathologic T stage	541				
T1&T2	350	Reference		Reference	
T3&T4	191	3.210 (2.373 - 4.342)	** <0.001**	1.619 (0.710 - 3.693)	0.252
Pathologic N stage	258				
N0	242	Reference		Reference	
N1	16	3.422 (1.817 - 6.446)	** <0.001**	1.503 (0.741 - 3.049)	0.259
Pathologic M stage	508				
M0	429	Reference		Reference	
M1	79	4.401 (3.226 - 6.002)	** <0.001**	2.867 (1.682 - 4.887)	** <0.001**
Pathologic stage	538				
Stage I&Stage II	332	Reference		Reference	
Stage III&Stage IV	206	3.910 (2.852 - 5.360)	** <0.001**	1.226 (0.480 - 3.129)	0.670
Histologic grade	533				
G1&G2	250	Reference		Reference	
G3&G4	283	2.665 (1.898 - 3.743)	** <0.001**	1.731 (1.059 - 2.829)	**0.029**
EPB41L2	541				
Low	270	Reference		Reference	
High	271	0.592 (0.434 - 0.807)	** <0.001**	0.564 (0.367 - 0.868)	**0.009**
Gender	541				
Female	187	Reference			
Male	354	0.924 (0.679 - 1.257)	0.613		
Race	534				
Asian&Black or African American	65	Reference			
White	469	1.233 (0.685 - 2.221)	0.485		
Age	541				
≤60	269	Reference		Reference	
> 60	272	1.791 (1.319 - 2.432)	** <0.001**	1.592 (1.034 - 2.452)	**0.035**
Pathologic T stage	541				
T1&T2	350	Reference		Reference	
T3&T4	191	3.210 (2.373 - 4.342)	** <0.001**	1.823 (0.785 - 4.234)	0.162
Pathologic N stage	258				
N0	242	Reference		Reference	
N1	16	3.422 (1.817 - 6.446)	** <0.001**	1.683 (0.833 - 3.397)	0.147
Pathologic M stage	508				
M0	429	Reference		Reference	
M1	79	4.401 (3.226 - 6.002)	** <0.001**	2.886 (1.679 - 4.960)	** <0.001**
Pathologic stage	538				
Stage I&Stage II	332	Reference		Reference	
Stage III&Stage IV	206	3.910 (2.852 - 5.360)	** <0.001**	1.017 (0.389 - 2.662)	0.972
Histologic grade	533				
G1&G2	250	Reference		Reference	
G3&G4	283	2.665 (1.898 - 3.743)	** <0.001**	1.589 (0.970 - 2.603)	0.066
EPB41L3	541				
Low	270	Reference		Reference	
High	271	0.679 (0.503 - 0.916)	**0.011**	0.645 (0.419 - 0.993)	**0.046**

The p-value of the bold indicator is less than 0.05, indicating statistical significance.

### Constructing a prognostic model integrating EPB41L and clinical pathological factors

**Figure 3.**
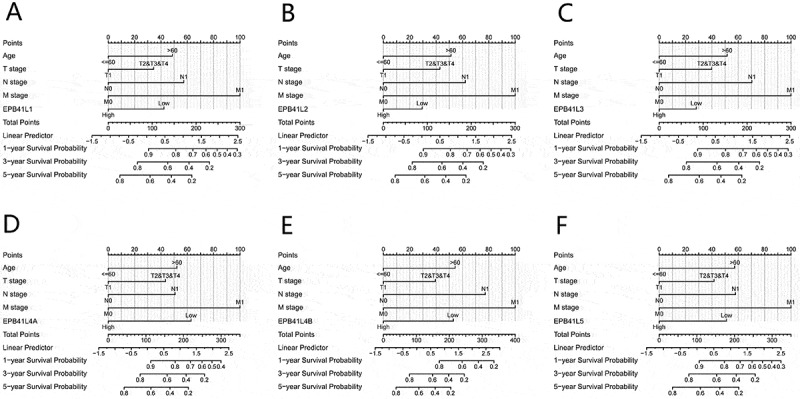
Nomogram for predicting 1-, 3-, and 5-year overall survival probabilities in KIRC patients. (A–F) This nomogram integrates EPB41L1-5 expression with additional prognostic factors derived from TCGA data.

A nomogram was developed that incorporates EPB41L1-5 expression along with independent clinical risk factors, including age and TNM stage (Figure 3(A–F)). Higher total points on the nomogram were associated with a worse prognosis.

### EPB41L-related functional enrichment analysis

**Figure 4.**
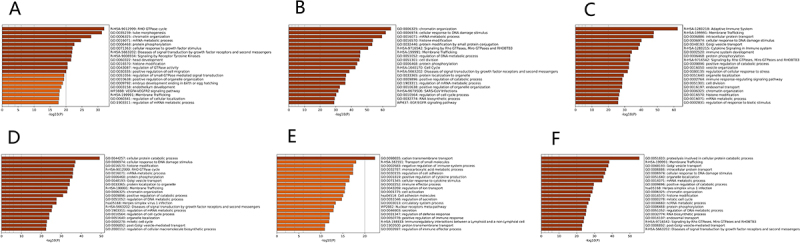
Functional Enrichment Analysis of EPB41L1-5 in KIRC. (A–F) Gene Ontology (GO) enrichment analysis and associated coexpression genes in Metascape. The GO terms are color-coded by p-value, with terms featuring a higher number of genes typically showing more significant p-values.

**Figure 5.**
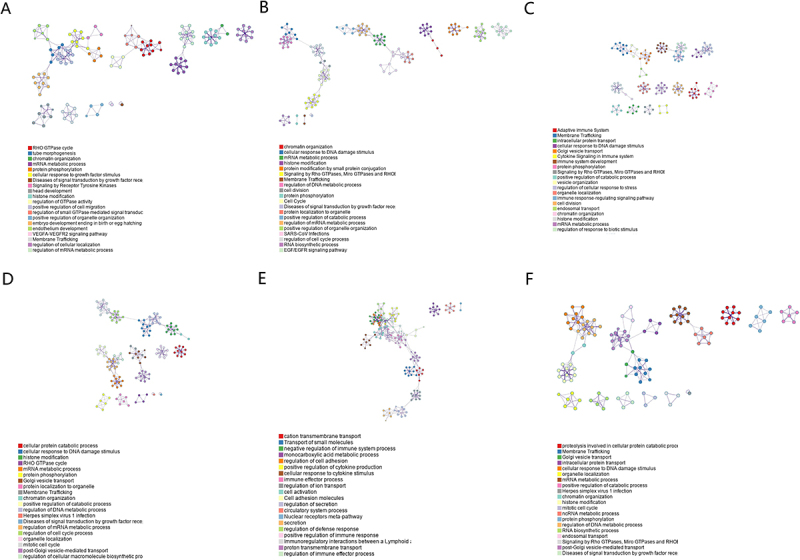
Interactive Network of Top Enrichment Terms. (A–F) the network is color-coded by cluster ID, with different colors representing various enrichment pathways associated with EPB41L1-5 correlated genes.

**Figure 6.**
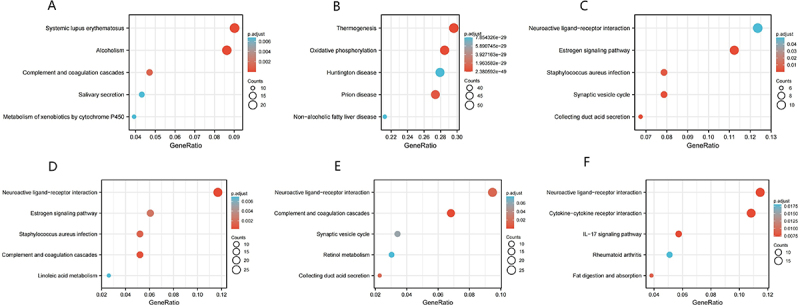
KEGG Enrichment Analysis for EPB41L1-5. (A–F) the plots illustrate the results of gene set enrichment analysis.

Gene enrichment analysis using GO identified a range of overrepresented terms across three primary functional categories: cellular component, biological process, and molecular function (Figure 4(A–F)). In the cellular component category, EPB41L and genes with similar expression patterns were primarily associated with the RHO GTPase cycle, mRNA metabolic processes, and membrane trafficking. Figure 5(A–F) presents an interactive network of the most significantly enriched terms. Additionally, KEGG functional enrichment analysis was performed using the clusterProfiler program, as shown in Figure 6(A–F).

### EPB41L -related signaling pathways obtained by GSEA

**Figure 7.**
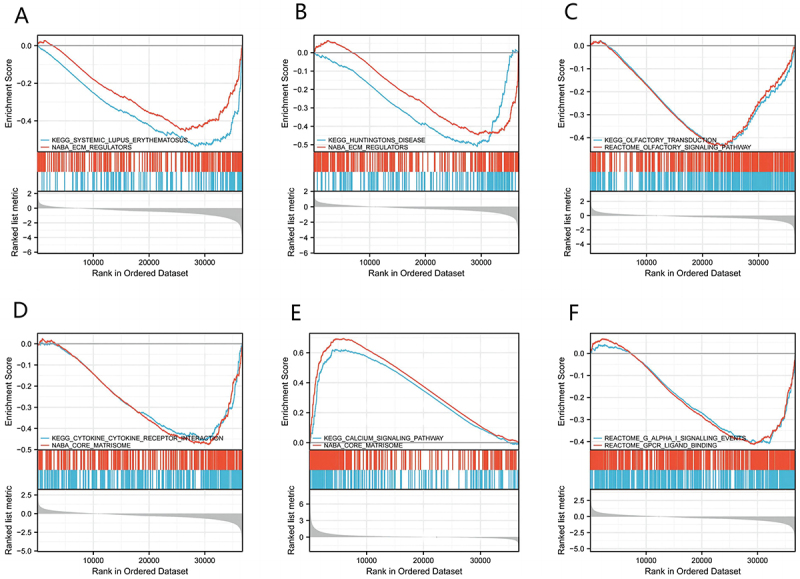
Functional Pathways of EPB41L1-5 in KIRC. (A–F) These panels depict pathways enriched in KIRC associated with EPB41L1-5.

To better understand the biological pathways associated with KIRC and their relationship with EPB41L expression levels, we compared RNA sequencing data from TCGA between high and low EPB41L1-5 expression groups. Through GSEA analysis of all differentially expressed genes, we identified several important signaling pathways related to EPB41L1-5, including extracellular matrix regulators, olfactory transduction, cytokine cytokine receptor interaction, core matrisome, and G-protein-coupled receptor ligand binding (Figure 7(A–F)).

### Genetic variations in EPB41L and their impact on OS

**Figure 8.**
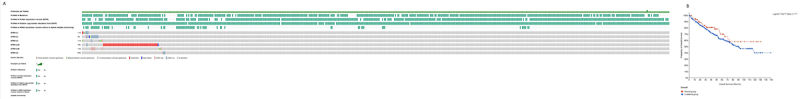
Genetic Alterations of EPB41L1-5 in KIRC. (A-B) This figure illustrates the genetic variations in EPB41L1-5 and their correlation with overall survival in KIRC patients.

We conducted an analysis of genetic modifications in EPB41L1-5 and their correlation with OS in KIRC. Figure 8(A,B) demonstrate a high incidence of EPB41L1-5 mutations in KIRC patients. The specific mutation rates for the six loci were 1.1%, 2%, 1.1%, 1.5%, 0.6%, and 10%, respectively. Although there is a higher likelihood of genetic mutations in EPB41L1-5, the association with OS in KIRC patients is limited.

### The relationship between EPB41L expression and immune cell infiltration

**Figure 9.**
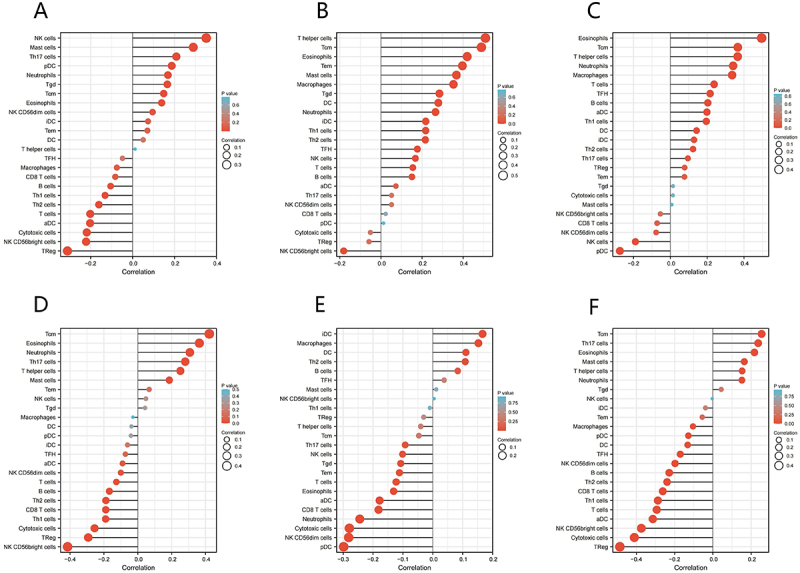
Correlation Between EPB41L1-5 Expression and Immune Infiltration in the KIRC Microenvironment. (A–F) the forest plot depicts the relationship between EPB41L1-5 expression levels and the infiltration of various immune cell subpopulations within the KIRC tumor microenvironment.

As shown in Figure 9(A–F) and Supplementary Table S3, there is a significant negative correlation between T cell infiltration and EPB41L1-5 expression. Additionally, in other subgroups, a correlation was observed between EPB41L expression and the counts of B cells, eosinophils, neutrophils, NK cells, Th1 cells, and Th2 cells.

### Relationship between EPB41L methylation level and prognosis

**Figure 10.**
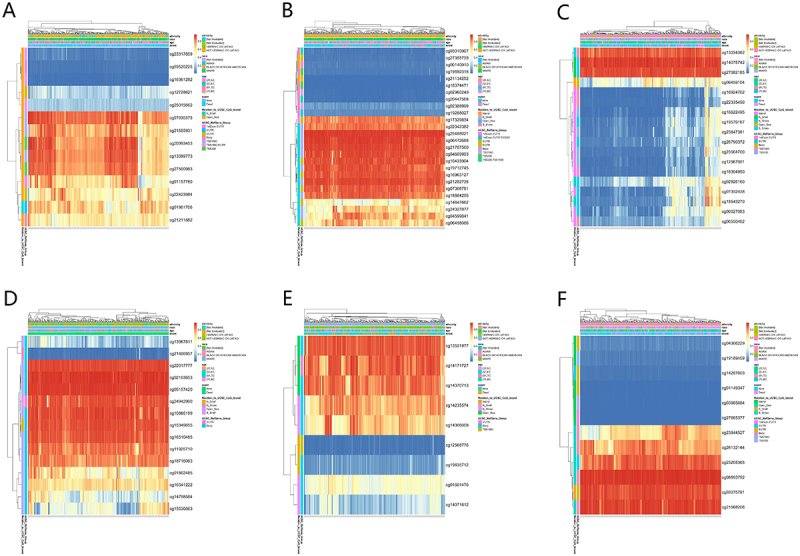
Methylation Patterns of EPB41L1-5 in KIRC. (A–F) Visualization of the relationship between EPB41L1-5 methylation levels and its expression.

**Figure 11.**
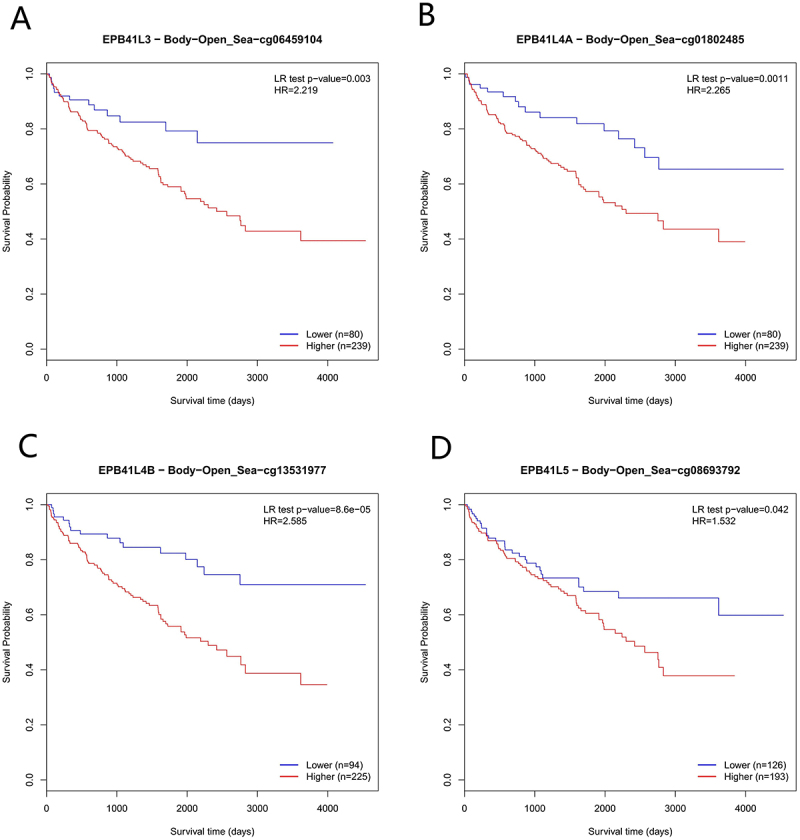
(A–D) Kaplan-Meier survival analysis based on the promoter methylation of EPB41L3-5.

According to the MethSurv analysis, EPB41L methylation levels were generally elevated in KIRC (Figures 10(A-F)). We evaluated the impact of methylation levels and EPB41L expression on the prognosis of KIRC and found that increased methylation levels were associated with poor prognosis in KIRC patients. Notably, high methylation levels at four CpG island sites (cg06459104, cg01802485, cg13531977, and cg08693792) were significantly correlated with adverse prognosis (as shown in Figures 11(A–D)).

### Reduced EPB41L expression in KIRC tissues and cell lines

**Figure 12.**
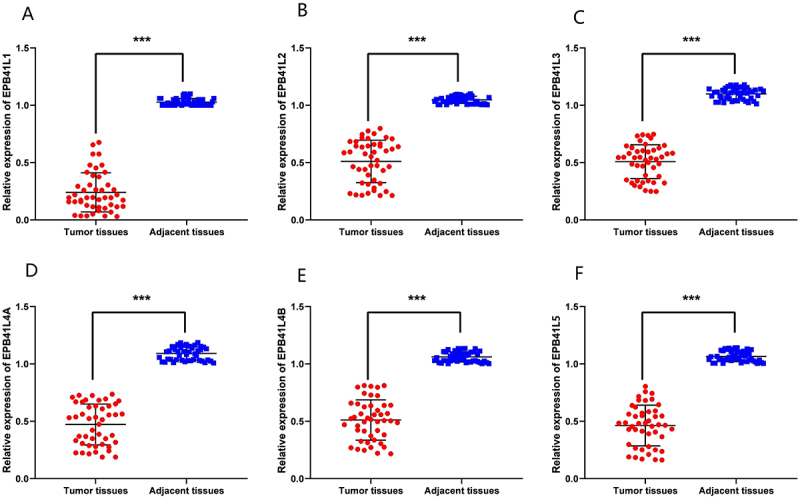
The expression of EPB41L mRNA levels in KIRC tissue and adjacent non-cancerous tissue is being investigated. (A–F) the mRNA expression levels of EPB41L1-5 are being compared in 47 clinical KIRC and adjacent non-cancerous tissue samples. ***p < .001.

**Figure 13.**
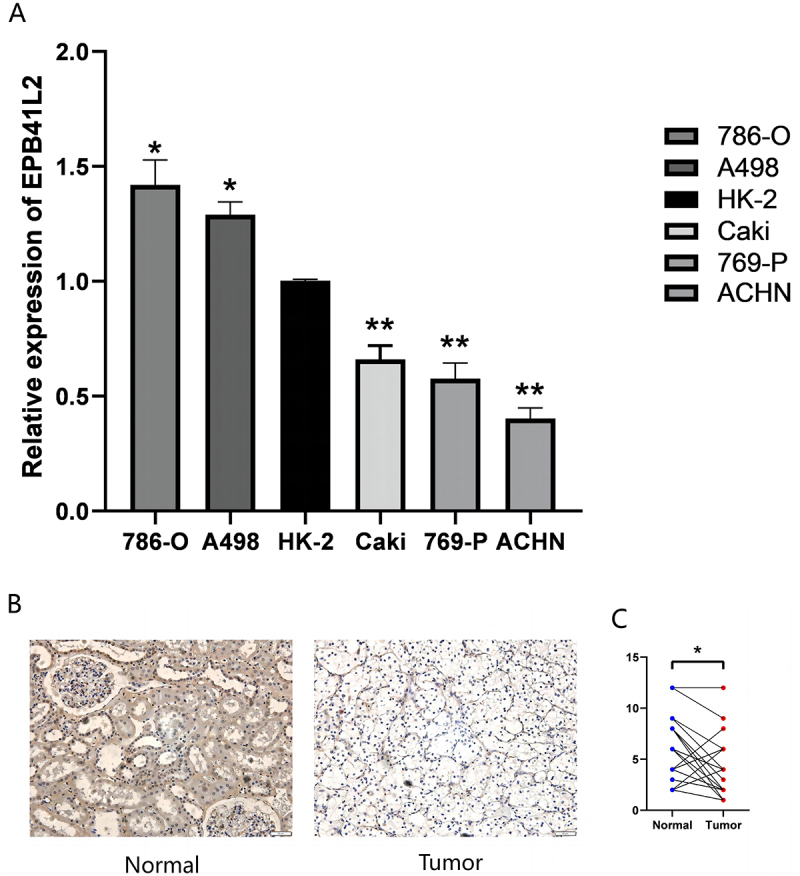
mRNA and protein expression levels of EPB41L2 in KIRC cell lines and tissues. (A) mRNA expression levels of EPB41L2 in KIRC cell lines. (B) EPB41L2 protein expression in KIRC tissues compared to normal renal tubular epithelial tissues. (C) Comparison of EPB41L2 expression between cancerous and adjacent non-cancerous tissues. *p < .05.

In the TCGA database, we have observed that the expression of EPB41L2 in KIRC tissue differs from that of other proteins in its family, as well as in normal tissue. However, the survival analysis trend remains consistent. To investigate this phenomenon, we conducted a series of experiments to validate the biological characteristics of EPB41L2 in KIRC. We obtained 47 pairs of tumor and adjacent tissues from KIRC patients for qRT-PCR validation. The research findings indicate a significant reduction in mRNA expression levels of all EPB41L genes, including EPB41L2, in tumor tissues (Figure 12). We further corroborated this discovery in renal cancer cell lines, where EPB41L2 mRNA was found to be low expressed in all renal cancer cell lines as compared to HK-2 (Figure 13(A)). The primer sequences employed have been presented in Table 3.

**Table 3.** Sequence of gene-specific primers for qPCR.

Gene	Forward sequence (5’− 3’)	Reverse sequence (5’− 3’)
EPB41L1	AGGAAACCACGCCGAGACACAA	GGTGGATGAGTTTGCTGTTGGG
EPB41L2	GTAAACGGGTCTCCAGGAGTCT	ACCACGGCAATGCTGACAAGTC
EPB41L3	ACCATGTCTCGCAGCTTGGATG	CTCCGTTTGTCCTCTTCCTCGT
EPB41L4A	CGAAGTTACCGCCAGTATCGCA	CAGAACCCTGAGCATCCGAAGA
EPB41L4B	GCAGTTGAGCACCACGCATTCT	CCTCTCAAAGGTGCTGGTTCTTC
EPB41L5	TCCTGTGCCAGTGGAGATAGAG	CGTTGCTTCCAATAAGTCTGGGC
GAPDH	GTCTCCTCTGACTTCAACAGCG	ACCACCCTGTTGCTGTAGCCAA

### EPB41L2 protein levels in KIRC tissue samples

To confirm the differential expression of EPB41L2 at the protein level, immunohistochemical staining was conducted on KIRC tissues and corresponding normal tissues. Figure 13(B) illustrates that the staining was primarily located in the cellular cytoplasm, with EPB41L2 expression notably reduced in KIRC tissues compared to normal tissues (Figure 13(C)).

### EPB41L2 expression levels in KIRC cells and construction of stable cell lines

**Figure 14.**
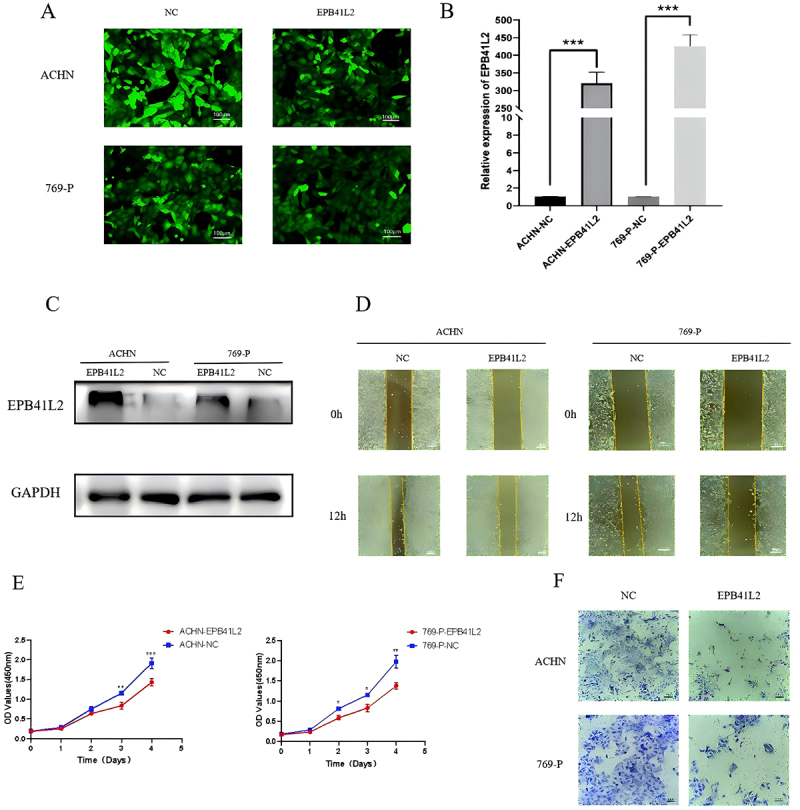
Lentiviral transfection of EPB41L2 in renal clear cell carcinoma cell lines and in vitro cell experiments. (A) the fluorescence transfection status of ACHN and 769-P KIRC cell lines after lentiviral transfection of EPB41L2 was observed for 72 hours. (B-C) After transduction with lentivirus overexpressing EPB41L2, the mRNA and protein expression levels of EPB41L2 in ACHN and 769-P were significantly elevated. (D) the proliferation of ACHN and 769-P cells was assessed by CCK-8 assay following overexpression of EPB41L2. (E) Scratch assay was performed to evaluate the migration of ACHN and 769-P cells overexpressing EPB41L2 for 12 hours. (F) Transwell invasion assay was conducted to investigate the alteration in invasive potential of ACHN and 769-P cells overexpressing EPB41L2. *p < .05, **p < .01, ***p < .001.

Using lentiviral transfection, this study established stable overexpression of EPB41L2 in KIRC cell lines ACHN and 769-P, as well as their corresponding control cell lines, named ACHN-EPB41L2, ACHN-NC, 769-P-EPB41L2, and 769-P-NC. After 72 hours of lentiviral transfection, the GFP fluorescence was observed under a fluorescence microscope, and the results showed that the transfection efficiency of the cells was approximately 80%, with normal cell morphology (Figure 14(A)). To validate the overexpression and knockdown efficiency of EPB41L2 in KIRC cell lines, this study extracted mRNA and protein from four groups of cells. The experimental results of qRT-PCR and Western blotting showed significant upregulation of EPB41L2 mRNA and protein in KIRC cell lines ACHN and 769-P after lentiviral transfection, with statistically significant differences (Figure 14(B-C)). These findings can be utilized for subsequent cellular functional experiments.

### EPB41L2 overexpression inhibits KIRC cell proliferation, migration, and invasion

This study conducted functional experiments on stable overexpression of EPB41L2 in KIRC cells ACHN and 769-P. The CCK-8 assay was used to detect cell proliferation at 0 h, 24 h, 48 h, and 72 h. The results showed that the absorbance of ACHN-EPB41L2 and 769-P-EPB41L2 cells at 48 h and 72 h was weaker than that of the negative control group, with statistically significant differences. This indicates that overexpression of EPB41L2 can significantly inhibit the proliferation ability of KIRC cells (Figure 14(E)). The scratch assay and Transwell invasion assay are effective methods for detecting cell migration and invasion ability. The scratch assay showed that compared with the negative control group, the migration rate of KIRC cell lines ACHN-EPB41L2 and 769-P-EPB41L2 overexpressing EPB41L2 was significantly reduced at 12 h, with statistically significant differences, suggesting that overexpression of EPB41L2 can inhibit the migration ability of KIRC cells (Figure 14(D)). Meanwhile, the results of the Transwell invasion assay showed that compared with the negative control group, the number of KIRC cells ACHN-EPB41L2 and 769-P-EPB41L2 overexpressing EPB41L2 that penetrated the basement membrane of the small chamber was lower, indicating that overexpression of EPB41L2 can inhibit the invasion ability of KIRC cells (Figure 14(F)).

## Discussion

The pathogenesis of KIRC remains incompletely understood. Currently, the main therapeutic targets for KIRC are inhibitors of vascular endothelial growth factor (VEGF) and its receptor (VEGFR), such as sunitinib and sorafenib [25,26]. However, these treatment modalities are limited by issues such as unstable efficacy and drug resistance [27]. Therefore, elucidating novel molecular mechanisms underlying KIRC is of critical importance for advancing therapeutic strategies. First, such insights will deepen our understanding of KIRC pathobiology and provide improved guidance for treatment decision-making. Second, the identification of innovative therapeutic targets may more effectively inhibit tumor growth and metastasis, thereby improving patient outcomes. Furthermore, the discovery of novel therapeutic targets has the potential to overcome the limitations of current treatment modalities.

EPB41L is a tumor suppressor gene that exhibits decreased expression or inactivation in various types of tumors [28–31]. Previous research indicates that EPB41L suppresses tumor cell proliferation and invasion through several mechanisms, including: Firstly, EPB41L can suppress the cell cycle progression, leading to cell cycle arrest at G1 phase and thus inhibiting cell proliferation [32]. Secondly, it can regulate multiple signaling pathways, such as Wnt, PI3K/Akt, and MAPK, affecting cell proliferation, apoptosis, migration, and invasion [33,34]. Thirdly, it can promote cell apoptosis, thereby inhibiting tumor cell proliferation [35]. Fourthly, EPB41L can modulate the cell cytoskeleton, affecting cell morphology and movement, and consequently influencing cell migration and invasion [29].

Our findings demonstrate that EPB41L expression is downregulated in KIRC tissues, supporting its potential involvement in KIRC pathogenesis. Key terms identified through GO enrichment analysis, including Rho GTPase activity, mRNA biosynthetic processes, cell cycle regulation, and mitotic events, suggest an association between EPB41L and aberrant cellular proliferation, cell division, and transcriptional regulation. Notably, Rho GTPases regulate multiple aspects of tumor cell behavior, including cytoskeletal dynamics, cell adhesion, and cell cycle progression, further implicating their role in tumor development [36]. Through cytoskeletal and RHO-GTPase – linked programs, EPB41L may influence chemokine secretion, receptor signaling, and extracellular-matrix architecture, thereby tuning CXCL9/10/11–CXCR3–mediated CD8 T-cell recruitment versus CXCL8–CXCR1/2–driven neutrophil and angiogenic programs, and CCL2/CCL5–CCR2/CCR5–mediated monocyte/Treg trafficking [37]. These shifts can alter Th1/Th2 balance and shape responsiveness to immune checkpoint inhibitors. GSEA revealed that EPB41L is involved in extracellular matrix regulation, cytokine-receptor interactions, and G-protein-coupled receptor ligand binding. EPB41L may exert its effects on tumorigenesis and disease progression through these pathways, ultimately influencing patient prognosis. Using nomogram analysis, we identified significant associations between age, TNM stage, and EPB41L expression with 1-, 3-, and 5-year overall survival probabilities in patients with KIRC. This model enables the generation of individualized prognostic scores for each patient, thereby enhancing the accuracy of survival prediction.

Immunotherapy represents a promising strategy for cancer treatment. The extent of T cell immune infiltration is closely linked to the effectiveness of immunotherapy, making it a focal point in current cancer immunotherapy research [38]. Th1 and Th2 are two distinct subtypes of T cells, each with a different role in tumors [39]. Th1 cells primarily secrete interferon-gamma (IFN-γ) and interleukin-2 (IL-2), which promote the activation and proliferation of immune effector cells, enhance T cell-mediated cytotoxicity, and inhibit tumor growth and metastasis. Conversely, Th2 cells predominantly produce IL-4, IL-5, and IL-13, which facilitate tumor cell survival and metastatic dissemination while attenuating T cell cytotoxic function, thereby promoting tumor progression. The Th1/Th2 balance plays a pivotal role in immune surveillance and tumor clearance [40–42]. In patients with KIRC, the ratio of Th1/Th2 cells in peripheral blood is significantly decreased, while the number of Th2 cells is significantly increased [43,44]. Studies have shown that the more severe the imbalance in Th1/Th2 cell ratio, the worse the prognosis of KIRC [45]. We observed that EPB41L15 expression associates with diverse immune infiltrates (T cells, B cells, NK cells, monocytes/macrophages, eosinophils, neutrophils), and Tcell infiltration is a determinant of immunotherapy benefit in KIRC. Together with the Th1/Th2 imbalance reported in KIRC, these data suggest that EPB41L may modulate immune contexture relevant to immune checkpoint inhibitors response.

Multiple studies have demonstrated a clear interaction between methylation and gene expression in renal cell carcinoma [46,47]. McRonald et al. have highlighted the abundance of DNA methylation in clear cell renal cell carcinoma, with these methylation sites primarily located in gene promoter regions and CpG islands [48]. The presence of these methylation sites can result in gene silencing or downregulation, thereby impacting tumor initiation and progression. For instance, the promoter region of the VHL gene contains a large number of methylation sites, resulting in downregulation of VHL gene expression and promoting tumor development [48,49]. We also examined the methylation status of EPB41L1-5 and found that hypermethylation was associated with poor clinical outcomes in KIRC. These findings suggest that EPB41L expression may be regulated through epigenetic mechanisms involving DNA methylation, providing an additional prognostic biomarker for KIRC.

Besides VHL, as an established biomarker, KIM-1 shows promise for noninvasive monitoring in renal disease and is being evaluated in KIRC as a urine/serum indicator of tumor burden and treatment response, enabling longitudinal surveillance that may detect subclinical progression and complement imaging [50]. In parallel, our manuscript indicates that the EPB41L family is broadly downregulated in KIRC and carries prognostic value linked to overall survival, immune infiltration, and methylation status. Conceptually, integrating a circulating marker like KIM-1 with tissue-based EPB41L readouts could enhance patient stratification: KIM-1 for real-time disease tracking, and EPB41L signatures for baseline risk and pathway insights. Such a composite panel may refine decisions on adjuvant therapy, follow-up intensity, and eligibility for immunotherapy or epigenetic strategies, contingent on prospective validation and standardized assay thresholds.

To validate the TCGA database findings, we performed qRT-PCR and immunohistochemistry on 45 paired KIRC tumor and adjacent normal tissue samples. The results demonstrated that EPB41L2 expression was significantly downregulated at both the mRNA and protein levels in KIRC tissues compared with adjacent normal tissues. The discrepancy between our experimental results and the database analysis may be attributable to the substantial imbalance in sample sizes between normal and tumor tissues in the TCGA repository, or to differences in analytical methodologies. To further elucidate the functional role of EPB41L2 in KIRC pathogenesis, we conducted a series of in vitro cellular assays. ACHN and 769-P cell lines, which exhibit relatively low endogenous EPB41L2 expression, were selected for functional experiments. Our results demonstrated that EPB41L2 functions as a tumor suppressor in KIRC cells.

Although our study enhances the understanding of the relationship between the EPB41L family and KIRC, several limitations should be acknowledged. First, the majority of data were derived from public databases, which precluded direct assessment of data quality. Further experimental validation is warranted to confirm our findings. Second, our functional studies were limited to in vitro cellular assays examining the tumor-suppressive properties of EPB41L2; the effects of EPB41L2 on tumor growth in vivo remain to be determined using animal models. Third, the precise mechanisms by which EPB41L suppresses KIRC progression remain to be elucidated. Future studies should delineate its contextspecific effects through targeted mechanistic experiments and extend these analyses to other renal cancer subtypes.

## Conclusion

In summary, EPB41L1–5 show consistent dysregulation and prognostic associations in KIRC. In vitro cytological experiments confirmed that EPB41L2 expression inhibits the malignant behavior of KIRC cells. This research offers a theoretical foundation for diagnosing, prognosticating, and selecting therapeutic targets for KIRC.

## Supplementary Material

supplementary figures and tables.docx

## Data Availability

Data will be made available by the corresponding author Hongwei Zhao upon reasonable request.

## References

[1] Siegel RL, Miller KD, Wagle NS, et al. Cancer statistics, 2023. CA Cancer J Clin. 2023;73(1):17–27. doi: 10.3322/caac.21763. Epub 2023/01/13. PubMed PMID: 36633525. 36633525

[2] Znaor A, Lortet-Tieulent J, Laversanne M, et al. International variations and trends in renal cell carcinoma incidence and mortality. Eur Urol. 2015;67(3):519–530. doi: 10.1016/j.eururo.2014.10.002. Epub 2014/12/03. PubMed PMID: 25449206. 25449206

[3] Barata PC, Rini BI. Treatment of renal cell carcinoma: current status and future directions. CA Cancer J Clin. 2017;67(6):507–524. doi: 10.3322/caac.21411. Epub 2017/09/30. PubMed PMID: 28961310. 28961310

[4] Conboy J, Kan YW, Shohet SB, et al. Molecular cloning of protein 4.1, a major structural element of the human erythrocyte membrane skeleton. Proc Natl Acad Sci U S A. 1986;83(24):9512–9516. doi: 10.1073/pnas.83.24.9512. Epub 1986/12/01. PubMed PMID: 3467321; PubMed Central PMCID: PMC387170. 3467321 PMC387170

[5] Walensky LD, Blackshaw S, Liao D, et al. A novel neuron-enriched homolog of the erythrocyte membrane cytoskeletal protein 4.1. J Neurosci. 1999;19(15):6457–6467. doi: 10.1523/jneurosci.19-15-06457.1999. Epub 1999/07/22. PubMed PMID: 10414974; PubMed Central PMCID: PMC6782826. 10414974 PMC6782826

[6] Liang T, Sang S, Shao Q, et al. Abnormal expression and prognostic significance of EPB41L1 in kidney renal clear cell carcinoma based on data mining. Cancer Cell Int. 2020;20(1):356. doi: 10.1186/s12935-020-01449-8. Epub 2020/08/08. PubMed PMID: 32760223; PubMed Central PMCID: PMC7393885. 32760223 PMC7393885

[7] Zeng R, Liu Y, Jiang ZJ, et al. EPB41L3 is a potential tumor suppressor gene and prognostic indicator in esophageal squamous cell carcinoma. Int J Oncol. 2018;52(5):1443–1454. doi: 10.3892/ijo.2018.4316. Epub 2018/03/24. PubMed PMID: 29568917; PubMed Central PMCID: PMC5873871. 29568917 PMC5873871

[8] Xi C, Ren C, Hu A, et al. Defective expression of protein 4.1N is correlated to tumor progression, aggressive behaviors and chemotherapy resistance in epithelial ovarian cancer. Gynecol Oncol. 2013;131(3):764–771. doi: 10.1016/j.ygyno.2013.08.015. Epub 2013/09/03. PubMed PMID: 23994105. 23994105

[9] Ciccarese C, Brunelli M, Montironi R, et al. The prospect of precision therapy for renal cell carcinoma. Cancer Treat Rev. 2016;49:37–44. doi: 10.1016/j.ctrv.2016.07.003. Epub 20160712. PubMed PMID: 27453294. 27453294

[10] Lai G, Zhong X, Liu H, et al. A novel m7G-related genes-based signature with prognostic value and predictive ability to select patients responsive to personalized treatment strategies in bladder cancer. Cancers (Basel). 2022;14(21):5346 doi: 10.3390/cancers14215346. Epub 2022/11/12. PubMed PMID: 36358764; PubMed Central PMCID: PMC9656096. 36358764 PMC9656096

[11] Lai G, Zhong X, Liu H, et al. Development of a hallmark pathway-related gene signature associated with immune response for lower grade gliomas. Int J Mol Sci. 2022;23(19):11971 doi: 10.3390/ijms231911971. Epub 2022/10/15. PubMed PMID: 36233273; PubMed Central PMCID: PMC9570050. 36233273 PMC9570050

[12] Lai G, Liu H, Deng J, et al. The characteristics of tumor microenvironment predict survival and response to immunotherapy in adrenocortical carcinomas. Cells. 2023;12(5):755 doi: 10.3390/cells12050755. Epub 2023/03/12. PubMed PMID: 36899891; PubMed Central PMCID: PMC10000893. 36899891 PMC10000893

[13] Ma Q, Tao H, Li Q, et al. OrganoidDB: a comprehensive organoid database for the multi-perspective exploration of bulk and single-cell transcriptomic profiles of organoids. Nucleic Acids Res. 2023;51(D1):D1086–d93. doi: 10.1093/nar/gkac942. Epub 2022/10/23. PubMed PMID: 36271792; PubMed Central PMCID: PMC9825539. 36271792 PMC9825539

[14] Gao C, Zhang R, Chen X, et al. Integrating internet multisource big data to predict the occurrence and development of COVID-19 cryptic transmission. NPJ Digit Med. 2022;5(1):161. doi: 10.1038/s41746-022-00704-8. Epub 2022/10/29. PubMed PMID: 36307547; PubMed Central PMCID: PMC9614204. 36307547 PMC9614204

[15] Schulz WA, Alexa A, Jung V, et al. Factor interaction analysis for chromosome 8 and DNA methylation alterations highlights innate immune response suppression and cytoskeletal changes in prostate cancer. Mol Cancer. 2007;6(1):14. doi: 10.1186/1476-4598-6-14. Epub 2007/02/07. PubMed PMID: 17280610; PubMed Central PMCID: PMC1797054. 17280610 PMC1797054

[16] Yang Q, Zhu L, Ye M, et al. Tumor suppressor 4.1N/EPB41L1 is epigenetic silenced by promoter methylation and MiR-454-3p in NSCLC. Front Genet. 2022;13:805960. doi: 10.3389/fgene.2022.805960. Epub 2022/07/08. PubMed PMID: 35795202; PubMed Central PMCID: PMC9251189. 35795202 PMC9251189

[17] Yang X, Han H, De Carvalho DD, et al. Gene body methylation can alter gene expression and is a therapeutic target in cancer. Cancer Cell. 2014;26(4):577–590. doi: 10.1016/j.ccr.2014.07.028. Epub 2014/09/30. PubMed PMID: 25263941; PubMed Central PMCID: PMC4224113. 25263941 PMC4224113

[18] Mootha VK, Lindgren CM, Eriksson KF, et al. PGC-1alpha-responsive genes involved in oxidative phosphorylation are coordinately downregulated in human diabetes. Nat Genet. 2003;34(3):267–273. doi: 10.1038/ng1180. Epub 2003/06/17. PubMed PMID: 12808457. 12808457

[19] Subramanian A, Tamayo P, Mootha VK, et al. Gene set enrichment analysis: a knowledge-based approach for interpreting genome-wide expression profiles. Proc Natl Acad Sci U S A. 2005;102(43):15545–15550. doi: 10.1073/pnas.0506580102. Epub 2005/10/04. PubMed PMID: 16199517; PubMed Central PMCID: PMC1239896. 16199517 PMC1239896

[20] Yu Y, Wang Z, Zheng Q, et al. FAM72 serves as a biomarker of poor prognosis in human lung adenocarcinoma. Aging (Albany NY). 2021;13(6):8155–8176. doi: 10.18632/aging.202625. Epub 2021/03/10. PubMed PMID: 33686947; PubMed Central PMCID: PMC8034972. 33686947 PMC8034972

[21] Gao J, Aksoy BA, Dogrusoz U, et al. Integrative analysis of complex cancer genomics and clinical profiles using the cBioPortal. Sci Signal. 2013;6(269):l1. doi: 10.1126/scisignal.2004088. Epub 2013/04/04. PubMed PMID: 23550210; PubMed Central PMCID: PMC4160307. PMC4160307 23550210

[22] Bindea G, Mlecnik B, Tosolini M, et al. Spatiotemporal dynamics of intratumoral immune cells reveal the immune landscape in human cancer. Immunity. 2013;39(4):782–795. doi: 10.1016/j.immuni.2013.10.003 Epub 2013/10/22. PubMed PMID: 24138885. 24138885

[23] Tang G, Sun K, Ding G, et al. Low expression of TSTD2 serves as a biomarker for poor prognosis in kidney renal clear cell carcinoma. Int J Gen Med. 2023;16:1437–1453. doi: 10.2147/ijgm.S408854 Epub 2023/04/28. PubMed PMID: 37114071; PubMed Central PMCID: PMC10126726. 37114071 PMC10126726

[24] Yao H, Lyu F, Ma J, et al. PIMREG is a prognostic biomarker involved in immune microenvironment of clear cell renal cell carcinoma and associated with the transition from G1 phase to S phase. Front Oncol. 2023;13:1035321. doi: 10.3389/fonc.2023.1035321 Epub 2023/02/14. PubMed PMID: 36776322; PubMed Central PMCID: PMC9909346. 36776322 PMC9909346

[25] Choueiri TK, Kaelin WG. Targeting the HIF2-VEGF axis in renal cell carcinoma. Nat Med. 2020;26(10):1519–1530. doi: 10.1038/s41591-020-1093-z Epub 2020/10/07. PubMed PMID: 33020645. 33020645

[26] Hsieh JJ, Purdue MP, Signoretti S, et al. Renal cell carcinoma. Nat Rev Disease Primers. 2017;3:17009. doi: 10.1038/nrdp.2017.9 Epub 2017/03/10. PubMed PMID: 28276433; PubMed Central PMCID: PMC5936048. 28276433 PMC5936048

[27] van der Mijn JC, Mier JW, Broxterman HJ, et al. Predictive biomarkers in renal cell cancer: insights in drug resistance mechanisms. Drug resistance updates: reviews and commentaries in antimicrobial and anticancer chemotherapy. Drug Resist Updates. 2014;17(4–6):77–88. doi: 10.1016/j.drup.2014.10.003 Epub 2014/12/03. PubMed PMID: 25457974. 25457974

[28] Feng G, Guo K, Yan Q, et al. Expression of protein 4.1 family in breast cancer: database mining for 4.1 family members in malignancies. Med Sci Monit. 2019;25:3374–3389. doi: 10.12659/msm.914085 Epub 2019/05/08. PubMed PMID: 31063460; PubMed Central PMCID: PMC6524556. 31063460 PMC6524556

[29] Schulz WA, Ingenwerth M, Djuidje CE, et al. Changes in cortical cytoskeletal and extracellular matrix gene expression in prostate cancer are related to oncogenic ERG deregulation. BMC Cancer. 2010;10(1):505. Epub 2010/09/24. doi: 10.1186/1471-2407-10-505 PubMed PMID: 20860828; PubMed Central PMCID: PMC2955608. 20860828 PMC2955608

[30] Yin X, Li G, Fan D, et al. Ehm2 transcript variant 1 inhibits breast cancer progression and increases E-cadherin stability. Carcinogenesis. 2022;43(12):1110–1120. doi: 10.1093/carcin/bgac076 Epub 2022/11/25. PubMed PMID: 36422008; PubMed Central PMCID: PMC10122424. 36422008 PMC10122424

[31] Zhang W, Lai R, He X, et al. Clinical prognostic implications of EPB41L4A expression in multiple myeloma. J Cancer. 2020;11(3):619–629. doi: 10.7150/jca.33805 Epub 2020/01/17. PubMed PMID: 31942185; PubMed Central PMCID: PMC6959044. 31942185 PMC6959044

[32] Kuns R, Kissil JL, Newsham IF, et al. Protein 4.1B expression is induced in mammary epithelial cells during pregnancy and regulates their proliferation. Oncogene. 2005;24(43):6502–6515. doi: 10.1038/sj.onc.1208813 Epub 2005/07/12. PubMed PMID: 16007173. 16007173

[33] Yang Q, Zhu M, Wang Z, et al. 4.1N is involved in a flotillin-1/β-catenin/wnt pathway and suppresses cell proliferation and migration in non-small cell lung cancer cell lines. Tumour Biol. 2016;37(9):12713–12723. doi: 10.1007/s13277-016-5146-3 Epub 2016/10/27. PubMed PMID: 27448302. 27448302

[34] Ye K, Hurt KJ, Wu FY, et al. Pike. A nuclear GTPase that enhances PI3kinase activity and is regulated by protein 4.1N. Cell. 2000;103(6):919–930. doi: 10.1016/s0092-8674(00)00195-1 Epub 2001/01/04. PubMed PMID: 11136977. 11136977

[35] Toporkiewicz M, Grzybek M, Meissner J, et al. Release of an ~55kDa fragment containing the actin-binding domain of β-spectrin by caspase-8 during FND-induced apoptosis depends on the presence of protein 4.1. Arch Biochem Biophys. 2013;535(2):205–213. doi: 10.1016/j.abb.2013.03.009 Epub 2013/04/13. PubMed PMID: 23578573. 23578573

[36] Woldu SL, Hutchinson RC, Krabbe LM, et al. The rho GTPase signalling pathway in urothelial carcinoma. Nat Rev Urol. 2018;15(2):83–91. doi: 10.1038/nrurol.2017.184 Epub 2017/11/15. PubMed PMID: 29133936. 29133936

[37] Santoni M, Bracarda S, Nabissi M, et al. CXC and CC chemokines as angiogenic modulators in nonhaematological tumors. Biomed Res Int. 2014;2014:768758. doi: 10.1155/2014/768758 Epub 20140529. PubMed PMID: 24971349; PubMed Central PMCID: PMC4058128. 24971349 PMC4058128

[38] Borst J, Ahrends T, Bąbała N, et al. Cd4(+) T cell help in cancer immunology and immunotherapy. Nat Rev Immunol. 2018;18(10):635–647. doi: 10.1038/s41577-018-0044-0 Epub 2018/07/31. PubMed PMID: 30057419. 30057419

[39] Thorsson V, Gibbs DL, Brown SD, et al. The immune landscape of cancer. Immunity. 2018;48(4):812–30.e14. doi: 10.1016/j.immuni.2018.03.023 Epub 2018/04/10. PubMed PMID: 29628290; PubMed Central PMCID: PMC5982584. 29628290 PMC5982584

[40] Matsuda A, Furukawa K, Takasaki H, et al. Preoperative oral immune-enhancing nutritional supplementation corrects Th1/Th2 imbalance in patients undergoing elective surgery for colorectal cancer. Dis Colon Rectum. 2006;49(4):507–516. doi: 10.1007/s10350-005-0292-5 Epub 2006/01/20. PubMed PMID: 16421661. 16421661

[41] Tosolini M, Kirilovsky A, Mlecnik B, et al. Clinical impact of different classes of infiltrating T cytotoxic and helper cells (Th1, Th2, Treg, Th17) in patients with colorectal cancer. Cancer Res. 2011;71(4):1263–1271. doi: 10.1158/0008-5472.Can-10-2907 Epub 2011/02/10. PubMed PMID: 21303976. 21303976

[42] Watt WC, Cecil DL, Disis ML. Selection of epitopes from self-antigens for eliciting Th2 or Th1 activity in the treatment of autoimmune disease or cancer. Semin Immunopathol. 2017;39(3):245–253. doi: 10.1007/s00281-016-0596-7 Epub 2016/12/16. PubMed PMID: 27975138. 27975138

[43] Li L, Yang C, Zhao Z, et al. Skewed T-helper (Th)1/2- and Th17/T regulatory‑cell balances in patients with renal cell carcinoma. Mol Med Rep. 2015;11(2):947–953. doi: 10.3892/mmr.2014.2778 Epub 2014/10/30. PubMed PMID: 25352158; PubMed Central PMCID: PMC4262517. 25352158 PMC4262517

[44] Tatsumi T, Kierstead LS, Ranieri E, et al. Disease-associated bias in T helper type 1 (Th1)/Th2 CD4(+) T cell responses against MAGE-6 in HLA-DRB10401(+) patients with renal cell carcinoma or melanoma. J Exp Med. 2002;196(5):619–628. doi: 10.1084/jem.20012142 Epub 2002/09/05. PubMed PMID: 12208877; PubMed Central PMCID: PMC2193999. 12208877 PMC2193999

[45] Onishi T, Ohishi Y, Goto H, et al. An assessment of the immunological status of patients with renal cell carcinoma based on the relative abundance of T-helper 1- and -2 cytokine-producing CD4+ cells in peripheral blood. BJU Int. 2001;87(9):755–759. doi: 10.1046/j.1464-410x.2001.02210.x Epub 2001/06/20. PubMed PMID: 11412209. 11412209

[46] Linehan WM, Ricketts CJ. The cancer genome atlas of renal cell carcinoma: findings and clinical implications. Nat Rev Urol. 2019;16(9):539–552. doi: 10.1038/s41585-019-0211-5 Epub 2019/07/07. PubMed PMID: 31278395. 31278395

[47] Sato Y, Yoshizato T, Shiraishi Y, et al. Integrated molecular analysis of clear-cell renal cell carcinoma. Nat Genet. 2013;45(8):860–867. doi: 10.1038/ng.2699 Epub 2013/06/26. PubMed PMID: 23797736. 23797736

[48] McRonald FE, Morris MR, Gentle D, et al. CpG methylation profiling in VHL related and VHL unrelated renal cell carcinoma. Mol Cancer. 2009;8(1):31. doi: 10.1186/1476-4598-8-31 Epub 2009/06/06. PubMed PMID: 19493342; PubMed Central PMCID: PMC2698845. 19493342 PMC2698845

[49] Yang W, Zhou J, Zhang Z, et al. Downregulation of lncRNA APCDD1L-AS1 due to DNA hypermethylation and loss of VHL protein expression promotes the progression of clear cell renal cell carcinoma. Int J Biol Sci. 2022;18(6):2583–2596. doi: 10.7150/ijbs.71519 Epub 2022/04/14. PubMed PMID: 35414787; PubMed Central PMCID: PMC8990466. 35414787 PMC8990466

[50] Rini BI, Albiges L, Tang X, et al. Circulating kidney injury molecule-1 (KIM-1) and association with outcome to adjuvant immunotherapy in renal cell carcinoma. Ann Oncol. 2025;36(12):1525–1534. doi: 10.1016/j.annonc.2025.08.007 Epub 20250828. PubMed PMID: 40885527. 40885527

