# Supplementary material for: EPB41L family serves as a prognostic biomarker for kidney renal clear cell carcinoma
Source: Cell Adh Migr. 2026 Feb 4;20(1):2624964. doi: 10.1080/19336918.2026.2624964 (PMC12885433; doi:10.1080/19336918.2026.2624964)
Supplement: supplementary figures and tables.docx [file KCAM_A_2624964_SM5824.docx]

**
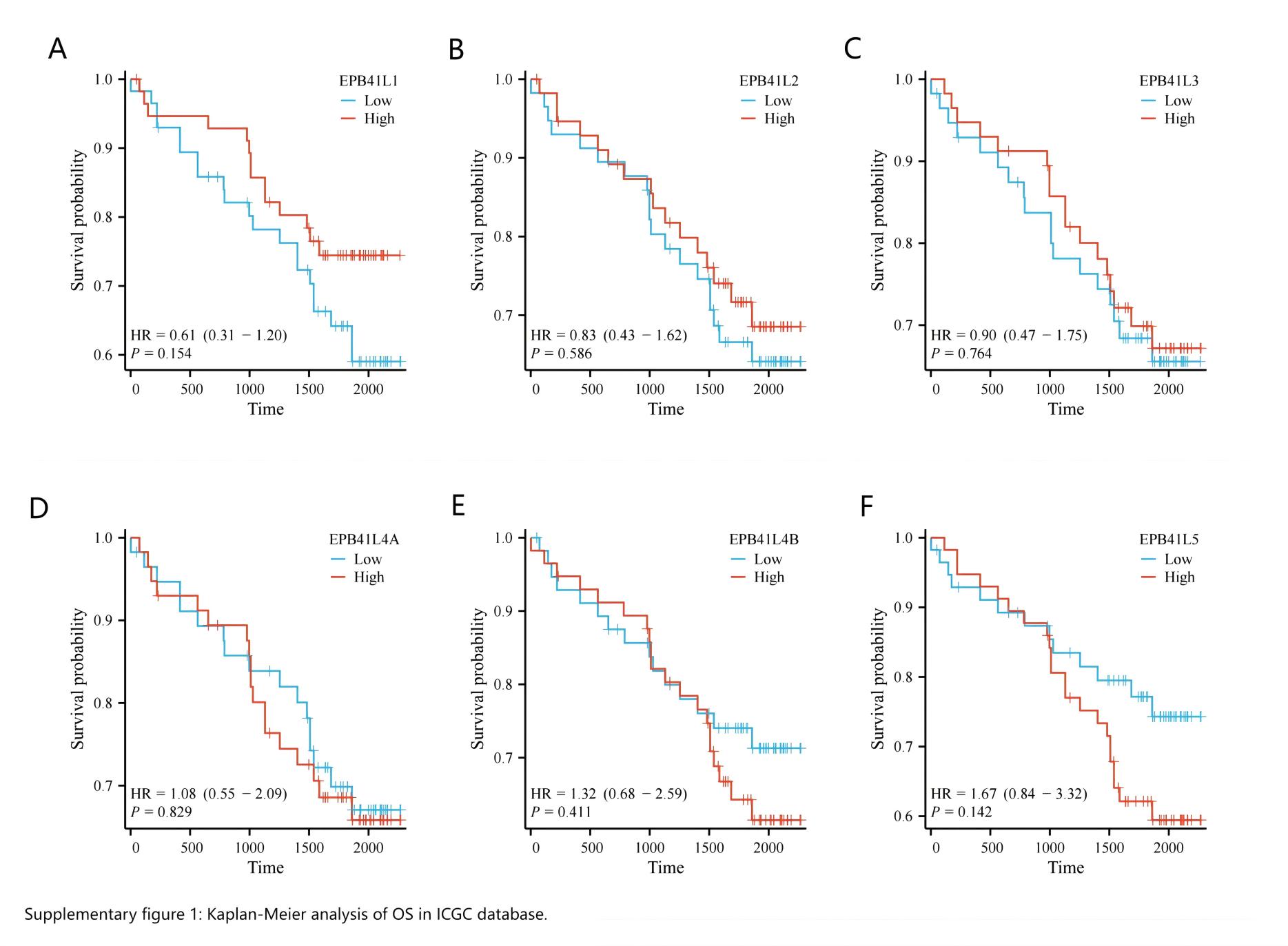
**

**
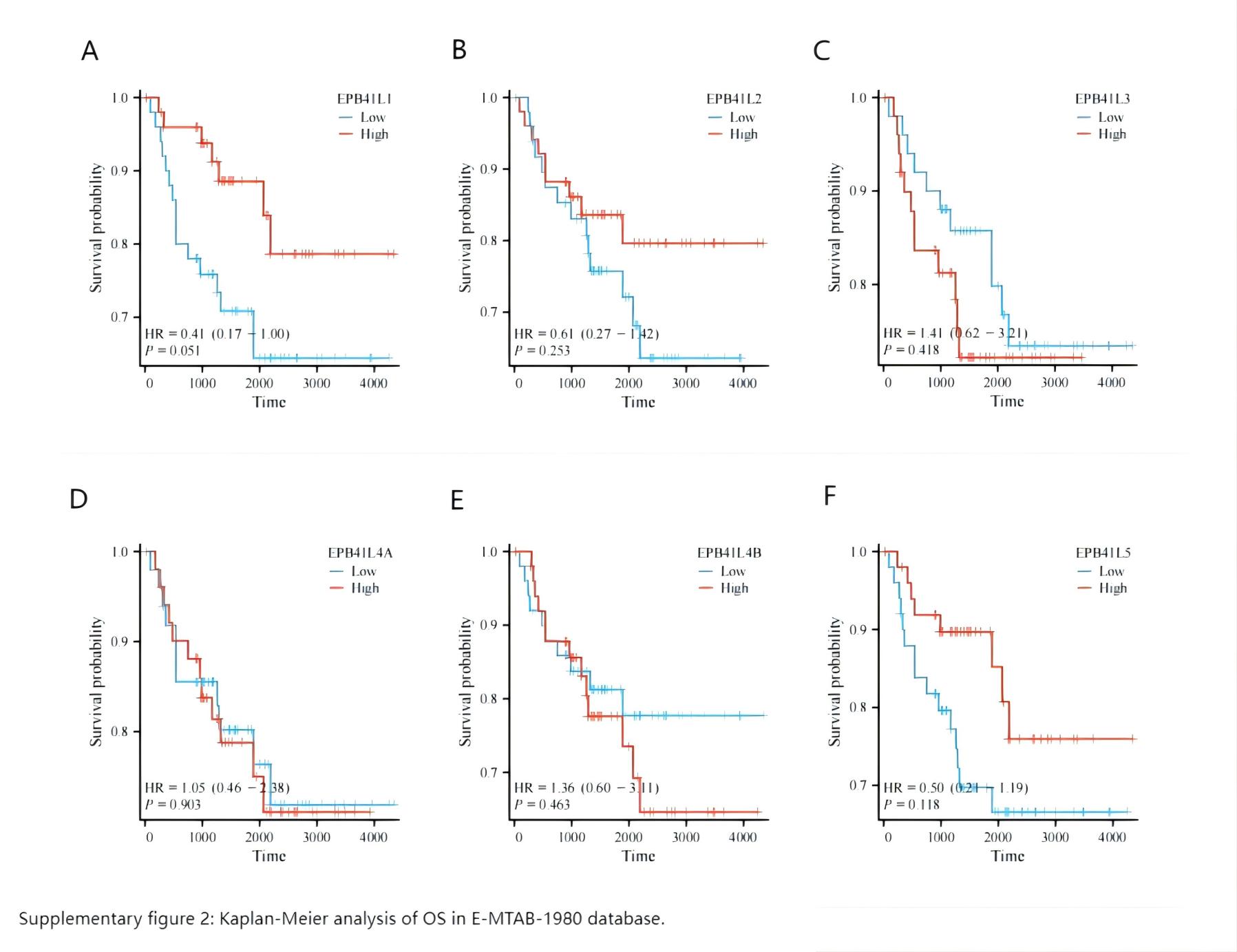
**

**Supplementary table 1. TCGA KIRC patient characteristics.**

| Characteristic | Low expression of EPB41L4A | High expression of EPB41L4A | p | Low expression of EPB41L4B | High expression of EPB41L4B | p | Low expression of EPB41L5 | High expression of EPB41L5 | p |
| --- | --- | --- | --- | --- | --- | --- | --- | --- | --- |
| n | 269 | 270 |  | 269 | 270 |  | 269 | 270 |  |
| T stage, n (%) |  |  | 0.009 |  |  | 0.051 |  |  | < 0.001 |
| T1 | 121 (22.4%) | 157 (29.1%) |  | 144 (26.7%) | 134 (24.9%) |  | 111 (20.6%) | 167 (31%) |  |
| T2 | 36 (6.7%) | 35 (6.5%) |  | 41 (7.6%) | 30 (5.6%) |  | 34 (6.3%) | 37 (6.9%) |  |
| T3 | 104 (19.3%) | 75 (13.9%) |  | 82 (15.2%) | 97 (18%) |  | 117 (21.7%) | 62 (11.5%) |  |
| T4 | 8 (1.5%) | 3 (0.6%) |  | 2 (0.4%) | 9 (1.7%) |  | 7 (1.3%) | 4 (0.7%) |  |
| N stage, n (%) |  |  | 0.068 |  |  | 0.238 |  |  | 0.003 |
| N0 | 116 (45.1%) | 125 (48.6%) |  | 120 (46.7%) | 121 (47.1%) |  | 112 (43.6%) | 129 (50.2%) |  |
| N1 | 12 (4.7%) | 4 (1.6%) |  | 5 (1.9%) | 11 (4.3%) |  | 14 (5.4%) | 2 (0.8%) |  |
| M stage, n (%) |  |  | 0.002 |  |  | 0.244 |  |  | < 0.001 |
| M0 | 199 (39.3%) | 229 (45.3%) |  | 215 (42.5%) | 213 (42.1%) |  | 204 (40.3%) | 224 (44.3%) |  |
| M1 | 52 (10.3%) | 26 (5.1%) |  | 33 (6.5%) | 45 (8.9%) |  | 55 (10.9%) | 23 (4.5%) |  |
| Age, median (IQR) | 62 (53, 70) | 59 (51, 69) | 0.119 | 60 (52, 70) | 61 (51.25, 70) | 0.843 | 61 (53, 70) | 60 (51, 69) | 0.292 |

**Supplementary table 2. Univariate and multivariate Cox regression model of prognosis for EPB41L4-5 in patients with KIRC**

| Characteristics | Total(N) | Univariate analysis | |  | Multivariate analysis | |
| --- | --- | --- | --- | --- | --- | --- |
|  |  | Hazard ratio (95% CI) | P value |  | Hazard ratio (95% CI) | P value |
| Gender | 541 |  |  |  |  |  |
| Female | 187 | Reference |  |  |  |  |
| Male | 354 | 0.924 (0.679 - 1.257) | 0.613 |  |  |  |
| Race | 534 |  |  |  |  |  |
| Asian&Black or African American | 65 | Reference |  |  |  |  |
| White | 469 | 1.233 (0.685 - 2.221) | 0.485 |  |  |  |
| Age | 541 |  |  |  |  |  |
| <= 60 | 269 | Reference |  |  | Reference |  |
| > 60 | 272 | 1.791 (1.319 - 2.432) | **< 0.001** |  | 1.655 (1.082 - 2.531) | **0.020** |
| Pathologic T stage | 541 |  |  |  |  |  |
| T1&T2 | 350 | Reference |  |  | Reference |  |
| T3&T4 | 191 | 3.210 (2.373 - 4.342) | **< 0.001** |  | 1.483 (0.653 - 3.365) | 0.346 |
| Pathologic N stage | 258 |  |  |  |  |  |
| N0 | 242 | Reference |  |  | Reference |  |
| N1 | 16 | 3.422 (1.817 - 6.446) | **< 0.001** |  | 1.479 (0.732 - 2.987) | 0.275 |
| Pathologic M stage | 508 |  |  |  |  |  |
| M0 | 429 | Reference |  |  | Reference |  |
| M1 | 79 | 4.401 (3.226 - 6.002) | **< 0.001** |  | 2.613 (1.547 - 4.414) | **< 0.001** |
| Pathologic stage | 538 |  |  |  |  |  |
| Stage I&Stage II | 332 | Reference |  |  | Reference |  |
| Stage III&Stage IV | 206 | 3.910 (2.852 - 5.360) | **< 0.001** |  | 1.297 (0.515 - 3.268) | 0.581 |
| Histologic grade | 533 |  |  |  |  |  |
| G1&G2 | 250 | Reference |  |  | Reference |  |
| G3&G4 | 283 | 2.665 (1.898 - 3.743) | **< 0.001** |  | 1.510 (0.913 - 2.496) | 0.108 |
| EPB41L4A | 541 |  |  |  |  |  |
| Low | 270 | Reference |  |  | Reference |  |
| High | 271 | 0.464 (0.338 - 0.636) | **< 0.001** |  | 0.563 (0.361 - 0.879) | **0.012** |
|  |  |  |  |  |  |  |
| Gender | 541 |  |  |  |  |  |
| Female | 187 | Reference |  |  |  |  |
| Male | 354 | 0.924 (0.679 - 1.257) | 0.613 |  |  |  |
| Race | 534 |  |  |  |  |  |
| Asian&Black or African American | 65 | Reference |  |  |  |  |
| White | 469 | 1.233 (0.685 - 2.221) | 0.485 |  |  |  |
| Age | 541 |  |  |  |  |  |
| <= 60 | 269 | Reference |  |  | Reference |  |
| > 60 | 272 | 1.791 (1.319 - 2.432) | **< 0.001** |  | 1.738 (1.130 - 2.675) | **0.012** |
| Pathologic T stage | 541 |  |  |  |  |  |
| T1&T2 | 350 | Reference |  |  | Reference |  |
| T3&T4 | 191 | 3.210 (2.373 - 4.342) | **< 0.001** |  | 1.687 (0.745 - 3.822) | 0.210 |
| Pathologic N stage | 258 |  |  |  |  |  |
| N0 | 242 | Reference |  |  | Reference |  |
| N1 | 16 | 3.422 (1.817 - 6.446) | **< 0.001** |  | 1.933 (0.966 - 3.867) | 0.062 |
| Pathologic M stage | 508 |  |  |  |  |  |
| M0 | 429 | Reference |  |  | Reference |  |
| M1 | 79 | 4.401 (3.226 - 6.002) | **< 0.001** |  | 2.817 (1.669 - 4.754) | **< 0.001** |
| Pathologic stage | 538 |  |  |  |  |  |
| Stage I&Stage II | 332 | Reference |  |  | Reference |  |
| Stage III&Stage IV | 206 | 3.910 (2.852 - 5.360) | **< 0.001** |  | 1.117 (0.440 - 2.836) | 0.817 |
| Histologic grade | 533 |  |  |  |  |  |
| G1&G2 | 250 | Reference |  |  | Reference |  |
| G3&G4 | 283 | 2.665 (1.898 - 3.743) | **< 0.001** |  | 1.433 (0.868 - 2.365) | 0.160 |
| EPB41L4B | 541 |  |  |  |  |  |
| Low | 270 | Reference |  |  | Reference |  |
| High | 271 | 1.743 (1.285 - 2.365) | **< 0.001** |  | 1.832 (1.187 - 2.829) | **0.006** |
|  |  |  |  |  |  |  |
| Gender | 541 |  |  |  |  |  |
| Female | 187 | Reference |  |  |  |  |
| Male | 354 | 0.924 (0.679 - 1.257) | 0.613 |  |  |  |
| Race | 534 |  |  |  |  |  |
| Asian&Black or African American | 65 | Reference |  |  |  |  |
| White | 469 | 1.233 (0.685 - 2.221) | 0.485 |  |  |  |
| Age | 541 |  |  |  |  |  |
| <= 60 | 269 | Reference |  |  | Reference |  |
| > 60 | 272 | 1.791 (1.319 - 2.432) | **< 0.001** |  | 1.686 (1.101 - 2.581) | **0.016** |
| Pathologic T stage | 541 |  |  |  |  |  |
| T1&T2 | 350 | Reference |  |  | Reference |  |
| T3&T4 | 191 | 3.210 (2.373 - 4.342) | **< 0.001** |  | 1.583 (0.697 - 3.597) | 0.273 |
| Pathologic N stage | 258 |  |  |  |  |  |
| N0 | 242 | Reference |  |  | Reference |  |
| N1 | 16 | 3.422 (1.817 - 6.446) | **< 0.001** |  | 1.557 (0.773 - 3.138) | 0.215 |
| Pathologic M stage | 508 |  |  |  |  |  |
| M0 | 429 | Reference |  |  | Reference |  |
| M1 | 79 | 4.401 (3.226 - 6.002) | **< 0.001** |  | 2.645 (1.563 - 4.476) | **< 0.001** |
| Pathologic stage | 538 |  |  |  |  |  |
| Stage I&Stage II | 332 | Reference |  |  | Reference |  |
| Stage III&Stage IV | 206 | 3.910 (2.852 - 5.360) | **< 0.001** |  | 1.159 (0.458 - 2.931) | 0.756 |
| Histologic grade | 533 |  |  |  |  |  |
| G1&G2 | 250 | Reference |  |  | Reference |  |
| G3&G4 | 283 | 2.665 (1.898 - 3.743) | **< 0.001** |  | 1.550 (0.938 - 2.562) | 0.087 |
| EPB41L5 | 541 |  |  |  |  |  |
| Low | 270 | Reference |  |  | Reference |  |
| High | 271 | 0.445 (0.324 - 0.610) | **< 0.001** |  | 0.635 (0.403 - 1.001) | 0.051 |

Supplementary Table 3. The association between the expression level of EPB41l1-5 and the

immune infiltration in the tumor microenvironment.

|  | Immune cell | correlation(Pearson) | P-value(Pearson) | correlation(Spearman) | P-value(Spearman) |
| --- | --- | --- | --- | --- | --- |
| EPB41L1 | aDC | -0.110 | 0.011 | -0.203 | <0.001 |
| EPB41L1 | B cells | -0.087 | 0.044 | -0.105 | 0.015 |
| EPB41L1 | CD8 T cells | 0.018 | 0.682 | -0.082 | 0.056 |
| EPB41L1 | Cytotoxic cells | -0.124 | 0.004 | -0.219 | <0.001 |
| EPB41L1 | DC | 0.092 | 0.034 | 0.051 | 0.239 |
| EPB41L1 | Eosinophils | 0.181 | <0.001 | 0.139 | 0.001 |
| EPB41L1 | iDC | 0.085 | 0.048 | 0.074 | 0.087 |
| EPB41L1 | Macrophages | -0.050 | 0.244 | -0.075 | 0.082 |
| EPB41L1 | Mast cells | 0.297 | <0.001 | 0.290 | <0.001 |
| EPB41L1 | Neutrophils | 0.209 | <0.001 | 0.169 | <0.001 |
| EPB41L1 | NK CD56bright cells | -0.139 | 0.001 | -0.222 | <0.001 |
| EPB41L1 | NK CD56dim cells | 0.160 | <0.001 | 0.096 | 0.026 |
| EPB41L1 | NK cells | 0.381 | <0.001 | 0.352 | <0.001 |
| EPB41L1 | pDC | 0.244 | <0.001 | 0.187 | <0.001 |
| EPB41L1 | T cells | -0.116 | 0.007 | -0.202 | <0.001 |
| EPB41L1 | T helper cells | 0.019 | 0.656 | 0.012 | 0.779 |
| EPB41L1 | Tcm | 0.134 | 0.002 | 0.148 | <0.001 |
| EPB41L1 | Tem | 0.099 | 0.021 | 0.071 | 0.100 |
| EPB41L1 | TFH | -0.025 | 0.563 | -0.048 | 0.263 |
| EPB41L1 | Tgd | 0.166 | <0.001 | 0.166 | <0.001 |
| EPB41L1 | Th1 cells | -0.024 | 0.572 | -0.131 | 0.002 |
| EPB41L1 | Th17 cells | 0.252 | <0.001 | 0.209 | <0.001 |
| EPB41L1 | Th2 cells | -0.192 | <0.001 | -0.161 | <0.001 |
| EPB41L1 | TReg | -0.144 | <0.001 | -0.311 | <0.001 |
| EPB41L2 | aDC | 0.132 | 0.002 | 0.073 | 0.092 |
| EPB41L2 | B cells | 0.180 | <0.001 | 0.151 | <0.001 |
| EPB41L2 | CD8 T cells | -0.004 | 0.920 | 0.023 | 0.591 |
| EPB41L2 | Cytotoxic cells | 0.036 | 0.407 | -0.051 | 0.237 |
| EPB41L2 | DC | 0.258 | <0.001 | 0.279 | <0.001 |
| EPB41L2 | Eosinophils | 0.448 | <0.001 | 0.420 | <0.001 |
| EPB41L2 | iDC | 0.205 | <0.001 | 0.218 | <0.001 |
| EPB41L2 | Macrophages | 0.405 | <0.001 | 0.353 | <0.001 |
| EPB41L2 | Mast cells | 0.353 | <0.001 | 0.368 | <0.001 |
| EPB41L2 | Neutrophils | 0.312 | <0.001 | 0.266 | <0.001 |
| EPB41L2 | NK CD56bright cells | -0.145 | <0.001 | -0.181 | <0.001 |
| EPB41L2 | NK CD56dim cells | 0.113 | 0.009 | 0.051 | 0.233 |
| EPB41L2 | NK cells | 0.138 | 0.001 | 0.168 | <0.001 |
| EPB41L2 | pDC | 0.050 | 0.247 | 0.013 | 0.770 |
| EPB41L2 | T cells | 0.229 | <0.001 | 0.155 | <0.001 |
| EPB41L2 | T helper cells | 0.534 | <0.001 | 0.509 | <0.001 |
| EPB41L2 | Tcm | 0.499 | <0.001 | 0.489 | <0.001 |
| EPB41L2 | Tem | 0.413 | <0.001 | 0.397 | <0.001 |
| EPB41L2 | TFH | 0.189 | <0.001 | 0.178 | <0.001 |
| EPB41L2 | Tgd | 0.265 | <0.001 | 0.285 | <0.001 |
| EPB41L2 | Th1 cells | 0.311 | <0.001 | 0.218 | <0.001 |
| EPB41L2 | Th17 cells | 0.022 | 0.610 | 0.052 | 0.232 |
| EPB41L2 | Th2 cells | 0.246 | <0.001 | 0.216 | <0.001 |
| EPB41L2 | TReg | 0.029 | 0.502 | -0.058 | 0.177 |
| EPB41L3 | aDC | 0.190 | <0.001 | 0.198 | <0.001 |
| EPB41L3 | B cells | 0.186 | <0.001 | 0.203 | <0.001 |
| EPB41L3 | CD8 T cells | -0.097 | 0.025 | -0.072 | 0.094 |
| EPB41L3 | Cytotoxic cells | 0.068 | 0.113 | 0.014 | 0.747 |
| EPB41L3 | DC | 0.153 | <0.001 | 0.142 | <0.001 |
| EPB41L3 | Eosinophils | 0.483 | <0.001 | 0.496 | <0.001 |
| EPB41L3 | iDC | 0.154 | <0.001 | 0.128 | 0.003 |
| EPB41L3 | Macrophages | 0.358 | <0.001 | 0.336 | <0.001 |
| EPB41L3 | Mast cells | 0.048 | 0.270 | 0.008 | 0.856 |
| EPB41L3 | Neutrophils | 0.315 | <0.001 | 0.341 | <0.001 |
| EPB41L3 | NK CD56bright cells | -0.021 | 0.629 | -0.054 | 0.208 |
| EPB41L3 | NK CD56dim cells | -0.040 | 0.350 | -0.078 | 0.072 |
| EPB41L3 | NK cells | -0.204 | <0.001 | -0.190 | <0.001 |
| EPB41L3 | pDC | -0.204 | <0.001 | -0.274 | <0.001 |
| EPB41L3 | T cells | 0.258 | <0.001 | 0.238 | <0.001 |
| EPB41L3 | T helper cells | 0.384 | <0.001 | 0.366 | <0.001 |
| EPB41L3 | Tcm | 0.383 | <0.001 | 0.366 | <0.001 |
| EPB41L3 | Tem | 0.097 | 0.025 | 0.076 | 0.077 |
| EPB41L3 | TFH | 0.211 | <0.001 | 0.216 | <0.001 |
| EPB41L3 | Tgd | 0.005 | 0.911 | 0.014 | 0.741 |
| EPB41L3 | Th1 cells | 0.226 | <0.001 | 0.195 | <0.001 |
| EPB41L3 | Th17 cells | 0.091 | 0.035 | 0.095 | 0.027 |
| EPB41L3 | Th2 cells | 0.121 | 0.005 | 0.123 | 0.004 |
| EPB41L3 | TReg | 0.061 | 0.157 | 0.077 | 0.073 |
| EPB41L4A | aDC | -0.064 | 0.135 | -0.090 | 0.036 |
| EPB41L4A | B cells | -0.155 | <0.001 | -0.168 | <0.001 |
| EPB41L4A | CD8 T cells | -0.173 | <0.001 | -0.189 | <0.001 |
| EPB41L4A | Cytotoxic cells | -0.179 | <0.001 | -0.255 | <0.001 |
| EPB41L4A | DC | -0.042 | 0.328 | -0.038 | 0.379 |
| EPB41L4A | Eosinophils | 0.371 | <0.001 | 0.363 | <0.001 |
| EPB41L4A | iDC | -0.039 | 0.363 | -0.061 | 0.155 |
| EPB41L4A | Macrophages | 0.008 | 0.848 | -0.029 | 0.502 |
| EPB41L4A | Mast cells | 0.218 | <0.001 | 0.186 | <0.001 |
| EPB41L4A | Neutrophils | 0.265 | <0.001 | 0.307 | <0.001 |
| EPB41L4A | NK CD56bright cells | -0.351 | <0.001 | -0.415 | <0.001 |
| EPB41L4A | NK CD56dim cells | -0.090 | 0.037 | -0.100 | 0.020 |
| EPB41L4A | NK cells | 0.013 | 0.771 | 0.048 | 0.270 |
| EPB41L4A | pDC | 0.027 | 0.534 | -0.041 | 0.342 |
| EPB41L4A | T cells | -0.061 | 0.159 | -0.127 | 0.003 |
| EPB41L4A | T helper cells | 0.284 | <0.001 | 0.251 | <0.001 |
| EPB41L4A | Tcm | 0.453 | <0.001 | 0.422 | <0.001 |
| EPB41L4A | Tem | 0.088 | 0.040 | 0.067 | 0.121 |
| EPB41L4A | TFH | -0.061 | 0.157 | -0.073 | 0.089 |
| EPB41L4A | Tgd | 0.031 | 0.479 | 0.042 | 0.326 |
| EPB41L4A | Th1 cells | -0.128 | 0.003 | -0.190 | <0.001 |
| EPB41L4A | Th17 cells | 0.332 | <0.001 | 0.280 | <0.001 |
| EPB41L4A | Th2 cells | -0.202 | <0.001 | -0.188 | <0.001 |
| EPB41L4A | TReg | -0.235 | <0.001 | -0.293 | <0.001 |
| EPB41L4B | aDC | -0.343 | <0.001 | -0.178 | <0.001 |
| EPB41L4B | B cells | -0.113 | 0.008 | 0.083 | 0.053 |
| EPB41L4B | CD8 T cells | -0.206 | <0.001 | -0.182 | <0.001 |
| EPB41L4B | Cytotoxic cells | -0.441 | <0.001 | -0.279 | <0.001 |
| EPB41L4B | DC | -0.106 | 0.014 | 0.111 | 0.010 |
| EPB41L4B | Eosinophils | -0.293 | <0.001 | -0.131 | 0.002 |
| EPB41L4B | iDC | 0.087 | 0.043 | 0.166 | <0.001 |
| EPB41L4B | Macrophages | -0.063 | 0.144 | 0.153 | <0.001 |
| EPB41L4B | Mast cells | -0.016 | 0.703 | 0.012 | 0.789 |
| EPB41L4B | Neutrophils | -0.425 | <0.001 | -0.244 | <0.001 |
| EPB41L4B | NK CD56bright cells | -0.062 | 0.153 | 0.004 | 0.920 |
| EPB41L4B | NK CD56dim cells | -0.412 | <0.001 | -0.282 | <0.001 |
| EPB41L4B | NK cells | -0.198 | <0.001 | -0.101 | 0.019 |
| EPB41L4B | pDC | -0.366 | <0.001 | -0.299 | <0.001 |
| EPB41L4B | T cells | -0.331 | <0.001 | -0.123 | 0.004 |
| EPB41L4B | T helper cells | -0.161 | <0.001 | -0.040 | 0.349 |
| EPB41L4B | Tcm | -0.120 | 0.005 | -0.047 | 0.279 |
| EPB41L4B | Tem | -0.183 | <0.001 | -0.111 | 0.010 |
| EPB41L4B | TFH | -0.059 | 0.172 | 0.038 | 0.378 |
| EPB41L4B | Tgd | -0.184 | <0.001 | -0.107 | 0.013 |
| EPB41L4B | Th1 cells | -0.267 | <0.001 | -0.010 | 0.820 |
| EPB41L4B | Th17 cells | -0.143 | <0.001 | -0.092 | 0.032 |
| EPB41L4B | Th2 cells | -0.022 | 0.618 | 0.109 | 0.012 |
| EPB41L4B | TReg | -0.251 | <0.001 | -0.031 | 0.479 |
| EPB41L5 | aDC | -0.321 | <0.001 | -0.314 | <0.001 |
| EPB41L5 | B cells | -0.252 | <0.001 | -0.228 | <0.001 |
| EPB41L5 | CD8 T cells | -0.272 | <0.001 | -0.262 | <0.001 |
| EPB41L5 | Cytotoxic cells | -0.400 | <0.001 | -0.410 | <0.001 |
| EPB41L5 | DC | -0.152 | <0.001 | -0.132 | 0.002 |
| EPB41L5 | Eosinophils | 0.192 | <0.001 | 0.217 | <0.001 |
| EPB41L5 | iDC | -0.028 | 0.521 | -0.039 | 0.372 |
| EPB41L5 | Macrophages | -0.112 | 0.009 | -0.104 | 0.016 |
| EPB41L5 | Mast cells | 0.180 | <0.001 | 0.164 | <0.001 |
| EPB41L5 | Neutrophils | 0.108 | 0.012 | 0.152 | <0.001 |
| EPB41L5 | NK CD56bright cells | -0.362 | <0.001 | -0.374 | <0.001 |
| EPB41L5 | NK CD56dim cells | -0.215 | <0.001 | -0.198 | <0.001 |
| EPB41L5 | NK cells | -0.053 | 0.217 | -0.004 | 0.935 |
| EPB41L5 | pDC | -0.108 | 0.012 | -0.128 | 0.003 |
| EPB41L5 | T cells | -0.282 | <0.001 | -0.293 | <0.001 |
| EPB41L5 | T helper cells | 0.170 | <0.001 | 0.153 | <0.001 |
| EPB41L5 | Tcm | 0.282 | <0.001 | 0.255 | <0.001 |
| EPB41L5 | Tem | -0.042 | 0.334 | -0.055 | 0.199 |
| EPB41L5 | TFH | -0.176 | <0.001 | -0.171 | <0.001 |
| EPB41L5 | Tgd | 0.014 | 0.749 | 0.043 | 0.313 |
| EPB41L5 | Th1 cells | -0.277 | <0.001 | -0.287 | <0.001 |
| EPB41L5 | Th17 cells | 0.231 | <0.001 | 0.237 | <0.001 |
| EPB41L5 | Th2 cells | -0.232 | <0.001 | -0.239 | <0.001 |
| EPB41L5 | TReg | -0.445 | <0.001 | -0.486 | <0.001 |
